# Supplementary material for: Birth incidence, deaths and hospitalisations of children and young people with Down syndrome, 1990–2015: birth cohort study
Source: BMJ Open. 2020 Apr 1;10(4):e033770. doi: 10.1136/bmjopen-2019-033770 (PMC7170621; doi:10.1136/bmjopen-2019-033770)
Supplement: Author's manuscript [file bmjopen-2019-033770.draft_revisions.pdf]

BMJ Open

BMJ Open is committed to open peer review. As part of this commitment we make the peer review history of every article we publish publicly available.

When an article is published we post the peer reviewers' comments and the authors' responses online. We also post the versions of the paper that were used during peer review. These are the versions that the peer review comments apply to.

The versions of the paper that follow are the versions that were submitted during the peer review process. They are not the versions of record or the final published versions. They should not be cited or distributed as the published version of this manuscript.

BMJ Open is an open access journal and the full, final, typeset and author-corrected version of record of the manuscript is available on our site with no access controls, subscription charges or pay-per-view fees (http://bmjopen.bmj.com).

If you have any questions on BMJ Open's open peer review process please email info.bmjopen@bmj.com

BMJ Open

Birth incidence, deaths, and hospitalisations of children and young people with Down syndrome, 1990-2015: birth cohort study

Journal:	*BMJ Open*
Manuscript ID	bmjopen-2019-033770
Article Type:	Research
Date Submitted by the Author:	22-Aug-2019

Complete List of Authors:	Hughes-McCormack, Laura; University of Glasgow, Institute of Health and Wellbeing<br>McGowan, Ruth; West of Scotland Regional Genetics Service<br>Pell, J. P.; Univ Glasgow<br>Mackay, Daniel; University of Glasgow<br>Henderson, Angela; University of Glasgow, Institute of Health and Wellbeing<br>O'Leary, Lisa; Edinburgh Napier University<br>Cooper, Sally-Ann; Glasgow University , Institute of Health and Wellbeing

Keywords:	Down syndrome, intellectual disabilities, births, incidence, deaths, hospitalisations

SCHOLARONE™
Manuscripts

BMJ

I, the Submitting Author has the right to grant and does grant on behalf of all authors of the Work (as defined in the below author licence), an exclusive licence and/or a non-exclusive licence for contributions from authors who are: i) UK Crown employees; ii) where BMJ has agreed a CC-BY licence shall apply, and/or iii) in accordance with the terms applicable for US Federal Government officers or employees acting as part of their official duties; on a worldwide, perpetual, irrevocable, royalty-free basis to BMJ Publishing Group Ltd ("BMJ") its licensees and where the relevant Journal is co-owned by BMJ to the co-owners of the Journal, to publish the Work in this journal and any other BMJ products and to exploit all rights, as set out in our licence.

The Submitting Author accepts and understands that any supply made under these terms is made by BMJ to the Submitting Author unless you are acting as an employee on behalf of your employer or a postgraduate student of an affiliated institution which is paying any applicable article publishing charge ("APC") for Open Access articles. Where the Submitting Author wishes to make the Work available on an Open Access basis (and intends to pay the relevant APC), the terms of reuse of such Open Access shall be governed by a Creative Commons licence – details of these licences and which Creative Commons licence will apply to this Work are set out in our licence referred to above.

Other than as permitted in any relevant BMJ Author's Self Archiving Policies, I confirm this Work has not been accepted for publication elsewhere, is not being considered for publication elsewhere and does not duplicate material already published. I confirm all authors consent to publication of this Work and authorise the granting of this licence.

For peer review only

Birth incidence, deaths, and hospitalisations of children and young people with Down syndrome, 1990-2015: birth cohort study

For peer review only

Laura Anne Hughes-McCormack

Ruth McGowan

J.P. Pell

Daniel Mackay

Angela Henderson

Lisa O'Leary

*Sally-Ann Cooper

***Correspondence:**

Institute of Health and Wellbeing, University of Glasgow

Mental Health and Wellbeing group, Administrative Building, Gartnavel Royal Hospital,

1055 Great Western Road, Glasgow, G12 0XH, UK.

Sally-Ann.Cooper@glasgow.ac.uk

For peer review only - http://bmjopen.bmj.com/site/about/guidelines.xhtml

Abstract

Objective: To investigate current Down syndrome live birth and death rates, and childhood hospitalisations, compared with peers.

Setting: General community, Scotland, UK

Participants: All live-births with Down syndrome, 1990-2015, identified via Scottish regional cytogenetic laboratories, were each age-gender-neighbourhood deprivation matched with 5 non-Down syndrome controls.

Primary outcome: Record-linkage to Scotland's hospital admissions and death data. Time to event analyses (Cox regressions) were used to compare time to first hospitalisation, and readmission, and linear regression to compare length of hospital stay, year at first admission, type of first admission. and first discharge where appropriate.

Results: 689/1,479 (46.6%) female and 769/1,479 (51.9%) male children/young people with Down syndrome were identified (1.0/1,000 births). 96 (6.5%) had died during the study period, 16 times more than controls. More of the Down syndrome group had at least one admission (1,159 [78.4%] versus 3,351 [51.9%]; adjusted HR=1.70 [1.59, 1.83]), and re-admissions (988 [66.8%] versus 1,700 [26.3%]; adjusted HR=2.37 [1.97, 2.85]). More of their admissions were emergencies (77.6% versus 71.9%; first emergency admission adjusted HR=2.54 [2.35, 2.75]), and of longer duration (4.80 days [SD=11.69] versus 1.30 days [SD=3.64]; adjusted Coef=2.01 [CI 1.79-2.24]). Admission rate increased over time for the Down syndrome group (from 75.4% to 81.2%), and decreased for the control group (from 63.3% to 43.1%).

Conclusions: We provide contemporaneous statistics on the live birth rate of babies with Down syndrome, and their childhood death rate. They require more hospital admissions, readmissions, and emergency admissions, and longer lengths of stays than their peers, which has received scant research attention in the past. This demonstrates the importance of statutory planning as well as informal support to families to avoid added problems in child development and family bonding over and above that brought by the intellectual disabilities associated with Down syndrome, and the carer burden that families cope with.

Key words: Down syndrome, intellectual disabilities, births, incidence, deaths, hospitalisations

For peer review only - http://bmjopen.bmj.com/site/about/guidelines.xhtml

Strengths and limitations of this study

- A whole country population-based study on Down syndrome, with comprehensive identification via all NHS Scottish Regional Genetics Centres.
- 25 year period of linked data.
- Account taken of the potential confounders of age, gender, and extent of neighbourhood deprivation.
- There was some missing control data, which might have related to migration out of Scotland which was not accounted for.
- The use of routinely collected hospital and death statistics has many advantages including large scale coverage, but there is likely to be a degree of coding inaccuracy which is not quantified.

For peer review only

For peer review only - http://bmjopen.bmj.com/site/about/guidelines.xhtml

Introduction

Improvements in health and social care and attitudes to disability has increased the survival of people with Down syndrome in recent decades.[1-6] In England and Wales, it was estimated that there were 37,090 people with Down syndrome in 2011,[7] but exact numbers are not reported. The National Down Syndrome Cytogenetic Register for England and Wales produces data on the estimated live-birth rate for Down syndrome, using assumptions on termination rates for those receiving a pre-natal diagnosis. In 2013, it reported an estimated rate of 1.0/1,000.[8] In the U.S.A., de Graaf et al[9] estimated live birth and population prevalence for Down syndrome for all 9 states in which data was available. They reported a live birth prevalence rate of 12.1/10,000, and an increase in the number of people living with Down syndrome from 1950 until 2010. In Canada, the live birth rate for Down syndrome was reported to have remained stable between 2005 and 2013, and higher than in the UK and USA rates, at approximately 13.5/10,000.[10] Similar data is not available for Scotland. Given the changing population demographic for people with Down syndrome, and limited evidence base, we require a better understanding of their live-birth rate and future health care needs. [4]

Down syndrome is associated with a range of congenital anomalies, particularly cardiac, and a higher risk for respiratory, immunological, endocrine, and gastrointestinal conditions.[4,11] This phenotype puts people with Down syndrome at risk of hospitalisation. This may impact on their development and family relationships, and so is an added disadvantage on top of their intellectual disabilities. Despite this, few previous studies have investigated hospitalisation of children and young people with Down syndrome, compared with the general population. Fitzgerald et al[12] studied hospital admissions of 405 children with Down syndrome born in Western Australia between 1983-2004. They found the children with Down

For peer review only - http://bmjopen.bmj.com/site/about/guidelines.xhtml

syndrome were hospitalised five times more often when compared with previously published general population data for the single year of 1995. They had no general population comparison data for length of stay. Zhu et al[13] investigated hospitalisations of 3,212 children and adults with Down syndrome compared to 67,204 children and adults without Down syndrome in Denmark, between 1977-2008. The people with Down syndrome had more than twice the rate of hospital admissions, and nearly three times as many bed days. Both studies found that length of admissions of the people with Down syndrome had reduced over the periods studied, and Zhu et al[13] reported more bed days for males, children under 5 years, and those with congenital heart anomalies. Other studies have lacked general population comparison data or been limited to single conditions. We are not aware of any large-scale studies specifically of children and young people with Down syndrome investigating these issues compared with their peers. There has been very little other investigation of the factors that influence length of hospital stay of children and young people with Down syndrome, and we located no studies on risk of readmission or on emergency admissions of children and young people.

Without accurate information on live-birth and death rates, and hospital admission needs, it is not possible to plan for the health care support that children with Down syndrome and their families need.

This study's aims were to investigate the (1) incidence of live births of people with Down syndrome over a 25 year period, (2) frequency of deaths of children and young people with Down syndrome compared to matched controls, and (3) hospital admission frequency and duration, emergency admissions, and readmissions of children and young people with Down syndrome compared to matched controls.

For peer review only

For peer review only - http://bmjopen.bmj.com/site/about/guidelines.xhtml

Methods

Ethical approval

Permission to access, link and analyse these data was granted by the Privacy Advisory Committee (PAC) to the National Health Service (NHS) National Services Scotland (NSS) and the Registrar General. Written informed consent was not obtained from participants for their clinical records to be used in this study, but patient records were anonymized and de-identified prior to analysis. Safehaven approval was granted to approved researchers to analyse the data.

Patient and Public Involvement

No patient involved.

Study sample, setting, and process

All NHS Scottish Regional Genetics Centres (the East, North, South-East and West Scotland Centres) identified all infants screened positive for Down syndrome (live births with trisomy 21, mosaic trisomy 21, or unbalanced translocation resulting in trisomy 21) between 01st January 1990 to 01st December 2015. In Scotland, everyone is given a unique Community Health Index (CHI) at birth which is used in their health records. The CHI database is held centrally by National Services Scotland (NSS), and was used to identify five controls without Down syndrome for each person with Down syndrome, matched on age (by month and year of birth), gender, and neighbourhood deprivation (using the Scottish Index of Multiple Deprivation: SIMD).[14] The CHI also provides a means to record link each person identified with Down syndrome and their matched controls to routinely collected hospital admissions data (Scottish Morbidity Records 01: SMR01),[15] and National Records of Scotland death

For peer review only - http://bmjopen.bmj.com/site/about/guidelines.xhtml

certificate data.[16] The end of the period of follow-up was 1st December 2015. SMR01 contains episode-based records for all non-psychiatric, non-obstetric acute hospital admissions in Scotland. The information collected includes the date of admission to hospital and discharge from it and whether the admission was routine or emergency. Data Quality Assurance Assessments for SMR01 are performed periodically (ISD Scotland, 2012; Turner et al, 2015).[17,18] National Records of Scotland (NRS) death certificate data include demographic information, and cause of death. The record is formed from the death certificates used to register the person's death. Cause of death coded using ICD-10.

Analyses

Incidence of Down syndrome: Down syndrome live-births were derived for each calendar year from 1990 to 2015 inclusive, and incidence calculated from all live-births in Scotland using data from National Records of Scotland births time series (NRS, 2019).[19]

Death: The fatality rate was described for people with Down syndrome and their matched controls.

Risk of any hospitalisation: Descriptive data on all-cause first hospital admissions was compared using dependent t tests for continuous variables and chi square tests for categorical variables. Time to all-cause first hospital admissions in the study period were then analysed using unconditional Cox regression models (log-log plots confirmed the proportional hazards assumption was met and censoring was (from date of birth) to date of first admission, date of death or end of study period). Down syndrome was entered as a binary yes/no, and year group (split into two groups 1990-2003 and 2004-2015), gender, extent of neighbourhood deprivation and admission type (emergency versus routine) were also entered as potential confounders.

For peer review only - http://bmjopen.bmj.com/site/about/guidelines.xhtml

Risk of emergency hospitalisation: Time to emergency first hospital admission in the study period were then analysed using unconditional Cox regression models in the whole population. Down syndrome was entered as a binary yes/no, and year group (split into two groups 1990-2003 and 2004-2015), gender and extent of neighbourhood deprivation were also entered as potential confounders. The follow up period was defined as from date of birth until date of first admission.

Duration of hospitalisation: Linear regression was used to model the duration of first hospital admission (log transformed to help negate potential skewing of results) as the outcome, with Down syndrome entered as a binary yes/no, and year group (split into two groups 1990-2003 and 2004-2015), gender, extent of neighbourhood deprivation and admission type (emergency versus routine) entered as potential confounders.

Risk of repeat hospitalisation: The time to first hospital re-admission of any type was analysed using unconditional Cox regression models. Down syndrome was entered as a binary yes/no, and year group (split into two groups 1990-2003 and 2004-2015), gender, extent of neighbourhood deprivation, admission type (emergency versus routine) and discharge type from first admission (i.e. regular discharge or irregular discharge [such as a patient discharging himself/herself against medical advice or death]) were entered as potential confounders and the interaction of group(Down Syndrome versus controls)*admission type(emergency versus routine) was included. The follow up period was defined as date of discharge from the first admission until date of the next admission.

Reference groups for all regression analyses were: not having Down syndrome, females, most affluent neighbourhoods, most recent year group (2004-2015), routine admissions (as opposed to emergency admissions), and regular type of hospital discharges (as opposed to

For peer review only - http://bmjopen.bmj.com/site/about/guidelines.xhtml

irregular types of hospital discharges including death). All analyses were conducted with IBM SPSS version 22.

Results

Between 01st January 1990 to 01st December 2015, the Scottish Regional Genetics Centres identified 1,479 live-births with Down syndrome. They were matched with 6,455 controls. 188 (12.7%) infants with Down syndrome could not be matched due to missing data, typically on neighbourhood deprivation (SIMD quintile), in view of missing post-code information, or missing CHI (e.g. migration out of Scotland). Hence the shortfall of 940 matches. Of the 1,479 infants born with Down syndrome, 689 (46.6%) were girls, 769 (51.9%) were boys, and sex was not recorded for 21 (1.4%). Of the 6,455 controls, 3,080 (47.7%) were girls, and 3,375 (52.3%) were boys.

Incidence of Down syndrome live-births over time

Given the total number of infants born was 1,508,483, the incidence of Down syndrome live-births was 1,479/1,508,483 (1.0/1,000 births). Table 1 and figure 1 show the incidence of Down syndrome live-births for each year of the study period. The birth rate in Scotland has fallen since the early 1990s, whilst the incidence of Down syndrome appears to have risen since the early 1990s.

Table 1: Incidence of Down syndrome by year of birth

Year of birth	Number of births registered in Scotland (all people)	Down Syndrome	
	N	N	(%)
1990	65,973	60	0.09%
1991	67,024	66	0.10%

For peer review only - http://bmjopen.bmj.com/site/about/guidelines.xhtml

1992	65,789	38	0.06%
1993	63,337	35	0.06%
1994	61,656	43	0.07%
1995	60,051	46	0.08%
1996	59,296	40	0.07%
1997	59,440	72	0.12%
1998	57,319	54	0.09%
1999	55,147	52	0.09%
2000	53,076	60	0.11%
2001	52,527	61	0.12%
2002	51,270	44	0.09%
2003	52,432	58	0.11%
2004	53,957	79	0.15%
2005	54,386	63	0.12%
2006	55,690	44	0.08%
2007	57,781	70	0.12%
2008	60,041	63	0.10%
2009	59,046	73	0.12%
2010	58,791	59	0.10%
2011	58,590	63	0.11%
2012	58,027	59	0.10%
2013	56,014	58	0.10%
2014	56,725	62	0.11%
2015	55,098	57	0.11%*
**Total**	**1,508,483**	**1,479**	**0.10%**

*In 2015 the Down syndrome total is for 11 months only, so the % in 2015 is adjusted to 11/12 months in the general population

Deaths

Of the 1,479 people with Down syndrome, 96 (6.5%) died during the study period (18,267 person years of follow up for the Down syndrome group), of whom 49 (51.0%) were female, 45 (46.9%) were male, and sex was not recorded for 2 (2.1%). Of the 6,455 matched controls, 23 (0.4%) died during the period (81,876 person years follow up for the controls), of whom 9 (39.1%) were females, and 14 (60.9%) were males. Death was therefore more than 16 times

For peer review only

For peer review only - http://bmjopen.bmj.com/site/about/guidelines.xhtml

more common in the Down syndrome group. The most common certified underlying causes of death for the Down syndrome group, by ICD10 code/main cause were congenital heart anomalies (n=33, 34.4%), Down syndrome (n=26, 27.1%) and infection (excludes respiratory infections) (n=11, 11.5%). This pattern was different compared to controls in whom causes of deaths were due to a range of other factors not found for people with Down syndrome (n=8, 34.8%), prematurity/perinatal causes (n=5, 21.7%) and congenital heart anomalies (n=5, 21.7%).

Admissions

Table 2 presents details on the first all-cause hospital admissions for the two groups. 1,159/1,479 (78.4%) of the Down syndrome group had at least one hospital admission compared to 3,351/6,455 (51.9%) of the control group (χ^2=346.994; df=1; P<0.001). Compared with the control group, the Down syndrome group were statistically younger at the time of first hospital admission (t(4529)=16.1, P<.001), had less admissions among females (χ^2=345.962; df=1; P<0.001), were more equally spread across neighbourhoods whilst the control group had a gradient across SIMD groups with those in the most deprived areas having more hospital admissions (χ^2=576.306; df=1; P<0.001). Compared with the control group, the Down syndrome group had more of their first hospital admissions as an emergency (χ^2=14.548; df=2; P=0.001), and had longer duration of admissions (4.80 days, SD=11.69 versus 1.30 days, SD=3.64) by 3.50 days (t(4513)=15.3, P<0.001). The rate of admissions increased over time for people with Down syndrome (from 550, 75.4% [in 1990-2003] to 609, 81.2% [in 2004-2015]) and decreased for controls (from 1,837, 63.3% [in 1990-2003] to 1,514, 43.1% [in 2004-2015]; t(22794)=-3.868, P<0.001). The gap between the higher rate of admissions has widened over time for people with Down syndrome compared to controls.

For peer review only - http://bmjopen.bmj.com/site/about/guidelines.xhtml

For peer review only

Table 2: First all-cause hospital admissions

		Down syndrome (n=1,159)	Control Group (n=3,351)	P-value+
		Mean(SD) or N(%)	Mean(SD) or N(%)	
Age at admission (years)		1.07 (SD=2.4)	3.33 (SD=4.5)	<0.001
Sex	Male	608 (52.5%)	1,873 (55.9%)	<0.001
	Female	551 (47.5%)	1,478 (44.1%)	
SIMD	Most affluent (9-10)	222 (19.2%)	575 (17.2%)	<0.001
	7-8	236 (20.4%)	619 (18.5%)	
	5-6	215 (18.6%)	602 (18.0%)	
	3-4	242 (20.9%)	732 (21.9%)	
	Most deprived (1-2)	241 (20.8%)	822 (24.5%)	
Emergency admission	Yes	899 (77.6%)	2,410 (71.9%)	0.001
	No	260 (22.4%)	941 (28.1%)	
Birth year group	Most recent	609 (81.2%)	1,514 (43.1%)	<0.001
	Less recent	550 (75.4%)	1,837 (63.3%)	
Duration of admission (days)		4.80 (SD=11.69)	1.30 (SD=3.64)	<0.001

Incl=including; SD=Standard deviation; SIMD=Scottish Index of Multiple Deprivation
Due to some missing data, SIMD denominators are for Down syndrome n=1,156/Control group n=3,350. Denominator for birth year group most recent/less recent are for Down syndrome n= 750/729/Control group n=3,515/2,900.

Table 3 displays the results of the Cox regressions for time to first all-cause hospital admission. The risk of admission was higher amongst people with Down syndrome compared to controls, with an unadjusted hazard ratio (HR)=1.72 (95% CI 1.60-1.84). This remained after adjusting for gender, year group, neighbourhood deprivation and type of first admission: HR=1.70 (95% CI 1.59-1.83).

Table 3: Risk for first all cause hospital admission

		Separately entered		Simultaneously entered	
		HR value	95% CI	HR value	95% CI
Group	No Down syndrome	ref	ref	ref	ref
	Down syndrome	1.715**	1.603-1.835	1.703**	1.589-1.826
Gender	Male	ref	ref	ref	ref
	Female	.924*	.871-.980	.966	.911-1.025
Year of birth	Most recent	ref	ref	ref	ref
	Least recent	.069**	0.63-.076	.085**	.078-.093
SIMD	Most affluent (9-10)	ref	ref	ref	ref
	7-8	1.044	.948-1.150	.999	.907-1.100
	5-6	.987	.895-1.088	.949	.861-1.047

For peer review only - http://bmjopen.bmj.com/site/about/guidelines.xhtml

	3-4	1.041	.948-1.144	.941	.857-1.034
	Most deprived (1-2)	1.085	.990-1.189	.942	.859-1.033
First admission type	Elective	ref	ref	ref	ref
	Emergency	1.724**	1.624-1.831	1.511**	1.420-1.607

*P<.05; **P<.001

Table 4 displays the results of the Cox regressions for time to first emergency hospital admission. The risk of emergency admission was higher amongst people with Down syndrome compared to controls, with an unadjusted hazard ratio (HR)=1.90 (95% CI 1.75-2.05). This remained after adjusting for gender, year group and neighbourhood deprivation: HR=2.54 (95% CI 2.35-2.75).

Table 4: Risk for first emergency hospital admission

		**Separately entered**		**Simultaneously entered**	
		**HR value**	**95% CI**	**HR value**	**95% CI**
Group	No Down syndrome	ref	ref	ref	ref
	Down syndrome	1.895**	1.753-2.048	2.540**	2.349-2.746
Gender	Male	ref	ref	ref	ref
	Female	1.063	.993-1.139	.938	.876-1.005
Year of birth	Most recent	ref	ref	ref	ref
	Least recent	.067**	.060-.074	.065**	.465-.539
SIMD	Most affluent (9-10)	ref	ref	ref	ref
	7-8	1.110	.992-1.242	1.116	.998-1.249
	5-6	1.051	.936-1.179	1.070	.953-1.201
	3-4	1.101	.987-1.230	1.105	.990-1.234
	Most deprived (1-2)	1.185	1.065-1.320	1.182*	1.062-1.316

*P<.05; **P<.001

Duration of hospitalisation

The mean duration of first hospital admission was significantly longer for the Down syndrome group at 4.80 days (SD=11.69) compared to the control group at 1.30 days (SD=3.64). After adjustment (with a log transformed time variable to account for any data skew) for year group at first admission, sex, neighbourhood deprivation and type of

For peer review only - http://bmjopen.bmj.com/site/about/guidelines.xhtml

admission (emergency vs routine), duration of hospital admission remained significantly longer for the Down syndrome group: Coef=2.01 (CI 1.79-2.24) (table 5).

Table 5: Duration of first all-cause hospital admission

		Separately entered		Simultaneously entered	
		**Coef**	**95% CI**	**Coef**	**95% CI**
Group	No Down syndrome	ref	ref	ref	ref
	Down syndrome	1.93**	1.71-2.14	2.01**	1.79-2.24
Gender	Female	ref	ref	ref	ref
	Male	-.280*	-.500-.059	-.214	-.433-.006
Year of birth	Most recent	ref	ref	ref	ref
	Least recent	-.011	-.233-.210	-.086	-.307-1.36
SIMD	Most affluent (9-10)	ref	ref	ref	ref
	7-8	.230	-.138-.599	.251	-.115-.617
	5-6	.406*	.032-.780	.416*	.045-.788
	3-4	.181	-.177-.539	.216	-.140-.527
	Most deprived (1-2)	.474*	.124-.823	.515*	.168-.862
First admission type	Elective	ref	ref	ref	ref
	Emergency	-.233	-.515-.069	.457*	.156-.757

*P<.05; **P<.001

Risk of re-admission

Of the Down syndrome group, 988/1,479 (66.8%) had at least one re-admission, compared to 1,700/6,455 (26.3%) of the control group (χ^2=147.970; df=1; P<0.001). The unadjusted HR of Down syndrome for time to readmission following discharge from first admission was 3.19 (95% CI 2.95-3.54). When adjusted for sex, year group at first admission, neighbourhood deprivation, type of admission, type of first discharge and group*emergency interaction, HR=2.37 (95% CI 1.97-2.85). Results are displayed in table 6.

Table 6: Time to readmission following discharge from first admission

		Separately entered		Simultaneously entered	
		**HR value**	**95% CI**	**HR value**	**95% CI**
Group	No Down syndrome	ref	ref	ref	ref
	Down syndrome	3.188**	2.946-3.541	2.371**	1.973-2.850

For peer review only - http://bmjopen.bmj.com/site/about/guidelines.xhtml

Gender	Male	ref	ref	ref	ref
	Female	.958	.888-1.034	.993	.919-1.073
Year of birth	Most recent	ref	ref	ref	ref
	Least recent	.523	.480-.569	.590**	.542-.641
SIMD	Most affluent (9-10)	ref	ref	ref	ref
	7-8	1.073	.944-1.219	.993	.873-1.130
	5-6	1.120	.985-1.274	1.069	.939-1.217
	3-4	1.147	1.014-1.443	1.084	.958-1.227
	Most deprived (1-2)	1.281	1.137-1.443	1.183*	1.049-1.335
First admission type	Elective	ref	ref	ref	ref
	Emergency	1.820**	1.652-2.004	1.680**	1.487-1.899
Discharge type	Routine	ref	ref	ref	ref
	Irregular	.209**	.183-.238	1.061	.925-1.218
Group*first admission type	-	-	-	1.109	.906-1.357

**P<.001

Discussion

Principal findings and interpretation

Over a 25 year period, we found that children/young people with Down syndrome compared with matched controls were considerably more likely to have had at least one hospital admission (78.4% compared to 51.9%; adjusted HR=1.70), and re-admissions (66.8% compared to 26.3%; adjusted HR=2.37). Their admissions were more likely to have been emergency admissions (77.6% compared to 71.9%; first emergency admission adjusted HR=2.54), and of longer duration (4.80 days compared to 1.30 days; adjusted Coef=2.01). Given the higher rate of first emergency admissions in the Down syndrome group, we included the interaction of group x type of first admission (emergency or routine) in the analysis of readmissions, but they still remained more likely to be readmitted. We are not aware of any previous study of readmissions and emergency admissions in this population. The rate of admissions increased over time for the children/young people with Down syndrome (75.4% to 81.2%), whilst it decreased for the control groups (from 63.3% to 43.1%). These findings are important, as they demonstrate considerable extra burden to both

For peer review only - http://bmjopen.bmj.com/site/about/guidelines.xhtml

For peer review only

children and young people with Down syndrome, and to their families. This demonstrates the importance of statutory as well as informal support to families to avoid added problems in child development over and above the intellectual disabilities that the children with Down syndrome experience, and the carer burden that families cope with. Additionally, the high rates of emergency admissions and re-admissions are suggestive of poorer quality management of health care at the primary care level (to avoid admissions), and have not to our knowledge been reported before.

The Down syndrome group were younger at the time of first hospital admission, had a more equal sex distribution for first admission than the control group, and an equal distribution across extent of neighbourhood deprivation, whilst the control group had a gradient with those in the most deprived areas more likely to have had a hospital admission. This highlights that findings in the general population cannot be relied upon when planning services for people with Down syndrome/intellectual disabilities, such as focussing services in areas of greatest deprivation, which would introduce inequalities within the population with Down syndrome. Families of children and young people with Down syndrome need focused support regardless of the areas they reside in.

We found the Scottish incidence of Down syndrome live-births to be 1.0/1,000 births over the last 25 years, with it being possibly higher now than in the early 1990s. The Scottish birth rate has fallen overall, but not for babies with Down syndrome. Death rate was 16 times higher for the children/young people with Down syndrome than for their controls, and their causes of death differed, including causes that might be expected to have been amenable to good health care such as infections.

For peer review only - http://bmjopen.bmj.com/site/about/guidelines.xhtml

For peer review only

Comparison with previous literature

As well as being 16 times more likely to die than their controls, we found that the children/young people with Down syndrome were more likely to be hospitalised, emergency hospitalised, re-admitted, and for longer durations. We have not identified any previous studies on re-admission with which to compare our findings, nor on emergency admissions. For the earlier period of 1977-2008 in Denmark, Zhu et al[13] reported that children and adults with Down syndrome had more than twice the rate of hospital admissions (all admissions, not separately reported for first admissions or repeat admissions), similar to our adjusted hazard ratios for first and repeat admissions, but we specifically investigated children and young people unlike that study. The smaller Australian study for the earlier period of 1983-2004 suggested the hospitalisation rate was five times higher, but relied on previously reported general population data from 1995.[12] As our study shows, and as previously reported in a study of children with Down syndrome identified from population-based sources in 1997 (n=210) and 2004 (n=208) in Western Australia,[20] it is highly likely that admission rates change over time, so these two studies are not strictly comparable to ours, and we have found no studies contemporaneous with ours with which to draw comparisons. Previous, small scale studies reported length of admissions of children with Down syndrome to be 4.7 days in a Swedish study of 211 children followed up for at least 17 years,[21] and 7 days specifically for bronchiolitis in a small retrospective case-control study in Singapore,[22] compared with the 4.8 days we found. A further small study of 213 children with Down syndrome aged 0-3 years in America reported high admission rates.[23]

The live birth incidence for Down syndrome we report is similar to that recently reported in England, USA, and Canada.[8-10] Antenatal screening changes may underlie the variability in the birth rates found over time. Previous research in Scotland showed that decisions to

For peer review only - http://bmjopen.bmj.com/site/about/guidelines.xhtml

terminate pregnancy following antenatal diagnosis of aneuploidy had fallen over time,[1] which our results are also suggestive of from the early 1990s to more recently. A range of complex factors influence women's decision-making regarding prenatal screening and diagnosis of Down syndrome, and it is possible that more societal acceptance of diversity and people with disabilities, or better availability of family support may influence decisions in Scotland over time,[1,24] although this requires further investigation.

Strengths and limitations

This is the first population-based study examining incidence of Down syndrome and health care utilisation over time in Scotland compared to the general population, and the first worldwide to investigate emergency hospital admissions and readmissions. It adjusted for potential confounders such as age, gender, SIMD. It was not subject to selection bias, as the population identification was based on routine data sources from all genetic service laboratories in Scotland (i.e. the whole population). The cox regression analysis for risk of admissions and readmissions provides advantages over logistic or linear techniques as this includes consideration of time to event (person time at risk). However, there was some missing control data, which might have related to migration out of Scotland which was not accounted for. The use of routinely collected hospital and death statistics has many advantages including large scale coverage, but there is likely to be a degree of coding inaccuracy which is not quantified.

Conclusions

As people with Down syndrome are living longer than in the past, we have provided contemporaneous statistics for the live birth rate of babies with Down syndrome and their childhood death rate compared with peers. We have found that their increasing survival is

For peer review only - http://bmjopen.bmj.com/site/about/guidelines.xhtml

not without health burden for the child, their family, and health services – we report the majority require hospital admissions, readmissions, and longer lengths of stays than their peers, which has received scant research attention in the past. Some factors may even suggest poorer health care than their peers, such as higher rates of emergency admissions. Together this brings important new findings necessary to address the challenge to plan support that children with Down syndrome and their families need. They need adequate health care, and also an awareness of the negative impact that hospitalisation can have on child development and family bonding. There may be a need to develop tailored packages of support for families with children/young people with Down syndrome to cope with the higher burden of health care needs of their offspring.

Footnotes

Contributors: LAH-M contributed to the study design, the analysis and interpretation of data, drafted the first article and revised it critically; DM, AH, LO contributed to study design, interpretation of data and revised the article critically; S-AC, JP, and RM conceived and designed the study, contributed to study analysis, interpretation of data and revised the article critically. All authors approved the final version

Funding: MRC Mental Health Data Pathfinder Award (MC_PC_17217), and the Scottish Government via the Scottish Learning Disabilities Observatory

Competing interests: The authors declare no competing interests

Ethical approval: Approval was gained from the Privacy Advisory Committee (PAC)

Word count: 3409

Data sharing statement: All data relevant to the study are included in the article or uploaded as supplementary information.

For peer review only - http://bmjopen.bmj.com/site/about/guidelines.xhtml

Reference

1. Jacobs M, Cooper S-A., McGowan R, Nelson S, Pell J. Pregnancy outcome following prenatal diagnosis of chromosomal anomaly: a record linkage study of 26,261 pregnancies. *PLoS ONE,* 2016; 11(12), e0166909.
2. Carothers AD, Boyd E, Lowther G, et al. Trends in prenatal diagnosis of Down syndrome and other autosomal trisomies in Scotland 1990 to 1994, with associated cytogenetic and epidemiological findings. *Genet Epidemiol.* 1999;16(2):179-90.
3. O'Leary L, Cooper SA, Hughes-McCormack L. Early death and causes of death of people with intellectual disabilities: A systematic review. *J Appl Res Intellect Disabil.* 2018;31(3):325-42.
4. Jensen KM, Bulova PD. Managing the care of adults with Down's syndrome. *BMJ.* 2014;349:g5596.
5. Hijii T, Fukushige J, Igarashi H, Takahashi N, Ueda K. Life expectancy and social adaptation in individuals with Down syndrome with and without surgery for congenital heart disease. *Clin Pediatr (Phila).* 1997;36(6):327-32.
6. Bull C, Rigby ML, Shinebourne EA. Should management of complete atrioventricular canal defect be influenced by coexistent Down syndrome. *Lancet.* 1985;1(8438):1147-9.
7. Wu J, Morris JK. The population prevalence of Down's syndrome in England and Wales in 2011. *Eur J Hum Genet.*2013;21(9):1016-9.
8. Morris, J.K., and Springett, A. (2014). The National Down Syndrome Cytogenetic Register for England and Wales: 2013 Annual Report. National Down Syndrome Cytogenetic Register. Queen Mary University of London.
9. de Graaf G, Buckley F, Skotko BG. Estimates of the live births, natural losses, and elective terminations with Down syndrome in the United States. *Am J Med Genet A.* 2015;167A(4):756-67.
10. Public Health Agency of Canada. Down syndrome surveillance in Canada, 2005-2013. Ottawa, 2017.
11. Ram G, Chinen J. Infections and immunodeficiency in Down syndrome. *Clin Exp Immunol.* 2011;164(1):9-16.
12. Fitzgerald P, Leonard H, Pikora TJ, Bourke J, Hammond G. Hospital admissions in children with down syndrome: experience of a population-based cohort followed from birth. *PLoS ONE.* 2013;8(8):e70401.
13. Zhu JL, Hasle H, Correa A, et al. Hospitalizations among people with Down syndrome: a nationwide population-based study in Denmark. *Am J Med Genet A.* 2013;161A(4):650-7.
14. National Statistics. Scottish Index of Multiple Deprivation 2012. Edinburgh: The Scottish Government; 2012.
15. Scottish Government, 2016: http://www.gov.scot/Topics/Statistics/SIMD)
16. Scottish Morbidity Records 01. http://www.ndc.scot.nhs.uk/Data-Dictionary/SMR-Datasets//SMR01-General-Acute-Inpatient-and-Day-Case/)
17. Information Services Division (ISD) Scotland: Assessment of SMR01 Data 2010-2011. Scotland Report; May 2012
18. Turner M, Barber M, Dodds H, Dennis M, Langhorne P, Macleod MJ. Agreement between routine electronic hospital discharge and Scottish Stroke Care Audit (SSCA) data in identifying stroke in the Scottish population. *BMC Health Serv Res*. 2015 Dec 30;15:583.
19. National Records of Scotland, (2019): https://www.nrscotland.gov.uk/statistics-and-data/statistics/statistics-by-theme/vital-events/births/births-time-series-data).

For peer review only - http://bmjopen.bmj.com/site/about/guidelines.xhtml

20. Thomas K, Bourke J, Girdler S, et al. Variation over time in medical conditions and health service utilization of children with Down syndrome. *J Pediatr*. 2011;158:194–200
21. Frid C, Anneren G, Rasmussen F, Sundelin C, Drott P. Utilization of medical care among children with Down's syndrome. *J Intellect Disabil Res*. 2002;46:310–317
22. Ting TW, Chan HY, Wong PPC, Testoni D, & Lee JH. Down syndrome increases hospital length of stay in children with bronchiolitis. *Proceedings of Singapore Healthcare*. 2016; *25*(1), 64–67.
23. So SA, Urbano RC, Hodapp RM. Hospitalizations of infants and young children with Down syndrome: evidence from inpatient person-records from a statewide administrative database. *J Intellect Disabil Res.* 2007;51(Pt 12):1030-8.
24. Olsen CL, Cross PK, Gensburg LJ. Down syndrome: interaction between culture, demography, and biology in determining the prevalence of a genetic trait. *Hum Biol.* 2003;75(4):503-20.

For peer review only - http://bmjopen.bmj.com/site/about/guidelines.xhtml

Figure 1: Incidence of Down syndrome births by year in Scotland from 1990-2015

For peer review only

For peer review only - http://bmjopen.bmj.com/site/about/guidelines.xhtml

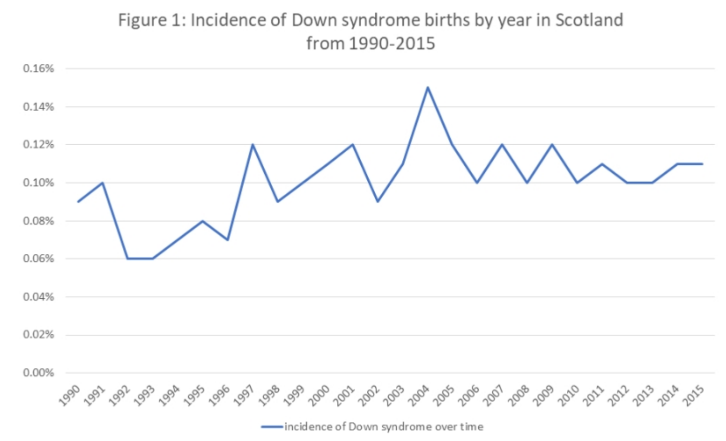

Figure 1: Incidence of Down syndrome births by year in Scotland from 1990-2015

338x190mm (96 x 96 DPI)

STROBE Statement—Checklist of items that should be included in reports of ***cohort studies***

	Item No	Recommendation	Page No
**Title and abstract**	1	(*a*) Indicate the study's design with a commonly used term in the title or the abstract<br>(*b*) Provide in the abstract an informative and balanced summary of what was done and what was found	2
**Introduction**			
Background/rationale	2	Explain the scientific background and rationale for the investigation being reported	4/5
Objectives	3	State specific objectives, including any prespecified hypotheses	5
**Methods**			
Study design	4	Present key elements of study design early in the paper	6
Setting	5	Describe the setting, locations, and relevant dates, including periods of recruitment, exposure, follow-up, and data collection	6-7
Participants	6	(*a*) Give the eligibility criteria, and the sources and methods of selection of participants. Describe methods of follow-up<br>(*b*) For matched studies, give matching criteria and number of exposed and unexposed	6-8
Variables	7	Clearly define all outcomes, exposures, predictors, potential confounders, and effect modifiers. Give diagnostic criteria, if applicable	6-8
Data sources/ measurement	8*	For each variable of interest, give sources of data and details of methods of assessment (measurement). Describe comparability of assessment methods if there is more than one group	6-8
Bias	9	Describe any efforts to address potential sources of bias	8
Study size	10	Explain how the study size was arrived at	6
Quantitative variables	11	Explain how quantitative variables were handled in the analyses. If applicable, describe which groupings were chosen and why	7-8
Statistical methods	12	(*a*) Describe all statistical methods, including those used to control for confounding<br>(*b*) Describe any methods used to examine subgroups and interactions<br>(*c*) Explain how missing data were addressed<br>(*d*) If applicable, explain how loss to follow-up was addressed<br>(*e*) Describe any sensitivity analyses	7-8
**Results**			
Participants	13*	(a) Report numbers of individuals at each stage of study—eg numbers potentially eligible, examined for eligibility, confirmed eligible, included in the study, completing follow-up, and analysed<br>(b) Give reasons for non-participation at each stage<br>(c) Consider use of a flow diagram	9-10
Descriptive data	14*	(a) Give characteristics of study participants (eg demographic, clinical, social) and information on exposures and potential confounders<br>(b) Indicate number of participants with missing data for each variable of interest<br>(c) Summarise follow-up time (eg, average and total amount)	9-14
Outcome data	15*	Report numbers of outcome events or summary measures over time	11-15

For peer review only

For peer review only - http://bmjopen.bmj.com/site/about/guidelines.xhtml

Main results	16	(*a*) Give unadjusted estimates and, if applicable, confounder-adjusted estimates and their precision (eg, 95% confidence interval). Make clear which confounders were adjusted for and why they were included<br>(*b*) Report category boundaries when continuous variables were categorized<br>(*c*) If relevant, consider translating estimates of relative risk into absolute risk for a meaningful time period	11-15
Other analyses	17	Report other analyses done—eg analyses of subgroups and interactions, and sensitivity analyses	11-15
**Discussion**			
Key results	18	Summarise key results with reference to study objectives	15-16
Limitations	19	Discuss limitations of the study, taking into account sources of potential bias or imprecision. Discuss both direction and magnitude of any potential bias	18
Interpretation	20	Give a cautious overall interpretation of results considering objectives, limitations, multiplicity of analyses, results from similar studies, and other relevant evidence	18-19
Generalisability	21	Discuss the generalisability (external validity) of the study results	18-19
**Other information**			
Funding	22	Give the source of funding and the role of the funders for the present study and, if applicable, for the original study on which the present article is based	19

*Give information separately for exposed and unexposed groups.

Note: An Explanation and Elaboration article discusses each checklist item and gives methodological background and published examples of transparent reporting. The STROBE checklist is best used in conjunction with this article (freely available on the Web sites of PLoS Medicine at http://www.plosmedicine.org/, Annals of Internal Medicine at http://www.annals.org/, and Epidemiology at http://www.epidem.com/). Information on the STROBE Initiative is available at http://www.strobe-statement.org.

For peer review only

For peer review only - http://bmjopen.bmj.com/site/about/guidelines.xhtml

BMJ Open

Birth incidence, deaths, and hospitalisations of children and young people with Down syndrome, 1990-2015: birth cohort study

Journal:	*BMJ Open*
Manuscript ID	bmjopen-2019-033770.R1
Article Type:	Original research
Date Submitted by the Author:	14-Jan-2020
Complete List of Authors:	Hughes-McCormack, Laura; University of Glasgow, Institute of Health and Wellbeing<br>McGowan, Ruth; West of Scotland Regional Genetics Service<br>Pell, J. P.; Univ Glasgow<br>Mackay, Daniel; University of Glasgow<br>Henderson, Angela; University of Glasgow, Institute of Health and Wellbeing<br>O'Leary, Lisa; Edinburgh Napier University<br>Cooper, Sally-Ann; Glasgow University , Institute of Health and Wellbeing
**Primary Subject Heading**:	Health services research
Secondary Subject Heading:	Health services research, Epidemiology
Keywords:	Down syndrome, intellectual disabilities, births, incidence, deaths, hospitalisations

SCHOLARONE™
Manuscripts

BMJ

I, the Submitting Author has the right to grant and does grant on behalf of all authors of the Work (as defined in the below author licence), an exclusive licence and/or a non-exclusive licence for contributions from authors who are: i) UK Crown employees; ii) where BMJ has agreed a CC-BY licence shall apply, and/or iii) in accordance with the terms applicable for US Federal Government officers or employees acting as part of their official duties; on a worldwide, perpetual, irrevocable, royalty-free basis to BMJ Publishing Group Ltd ("BMJ") its licensees and where the relevant Journal is co-owned by BMJ to the co-owners of the Journal, to publish the Work in this journal and any other BMJ products and to exploit all rights, as set out in our licence.

The Submitting Author accepts and understands that any supply made under these terms is made by BMJ to the Submitting Author unless you are acting as an employee on behalf of your employer or a postgraduate student of an affiliated institution which is paying any applicable article publishing charge ("APC") for Open Access articles. Where the Submitting Author wishes to make the Work available on an Open Access basis (and intends to pay the relevant APC), the terms of reuse of such Open Access shall be governed by a Creative Commons licence – details of these licences and which Creative Commons licence will apply to this Work are set out in our licence referred to above.

Other than as permitted in any relevant BMJ Author's Self Archiving Policies, I confirm this Work has not been accepted for publication elsewhere, is not being considered for publication elsewhere and does not duplicate material already published. I confirm all authors consent to publication of this Work and authorise the granting of this licence.

For peer review only

Birth incidence, deaths, and hospitalisations of children and young people with Down syndrome, 1990-2015: birth cohort study

Laura Anne Hughes-McCormack

Ruth McGowan

Jill P. Pell

Daniel Mackay

Angela Henderson

Lisa O'Leary

*Sally-Ann Cooper

***Correspondence:**

Institute of Health and Wellbeing, University of Glasgow

Mental Health and Wellbeing group, Administrative Building, Gartnavel Royal Hospital,

1055 Great Western Road, Glasgow, G12 0XH, UK.

Sally-Ann.Cooper@glasgow.ac.uk

For peer review only

Abstract

Objective: To investigate current Down syndrome live birth and death rates, and childhood hospitalisations, compared with peers.

Setting: General community.

Participants: All live-births with Down syndrome, 1990-2015, identified via Scottish regional cytogenetic laboratories, each age-gender-neighbourhood deprivation matched with 5 non-Down syndrome controls.

Primary outcome: Record-linkage to Scotland's hospital admissions and death data. Time to event analyses (Cox regressions) compared time to first hospitalisation, and readmission, and linear regression compared length of hospital stay, year at first admission, type of first admission, and first discharge.

Results: 689/1,479 (46.6%) female and 769/1,479 (51.9%) male children/young people with Down syndrome were identified (1.0/1,000 births, with no reduction over time); 1,291 were matched. 92/1,291 (7.1%) died during the period, 18 times more than controls. More of the Down syndrome group had at least one admission (1,130 [87.5%] versus 3,351 [51.9%]; adjusted HR=1.84 [1.68, 2.01]), and re-admissions (963 [85.2%] versus 1,700 [50.2%]; adjusted HR=2.55 [2.08, 3.14]). More of their admissions were emergencies (78.1% versus 71.6%; first emergency admission adjusted HR=2.87 [2.61, 3.15]), and longer duration (5.03 days [Median=2], versus 1.78 days [Median=1]) by 3.25 days; adjusted (log transformed) Coefficient=0.25 (CI 0.22-0.28). Admission rate decreased from 1990-2003 to 2004-2015 for the Down syndrome group (90.7% to 84.9%), and for controls (63.3% to 43.0%).

Conclusions: We provide contemporaneous statistics on the live birth rate of babies with Down syndrome, and their childhood death rate. They require more hospital admissions, readmissions, and emergency admissions, and longer lengths of stays than their peers, which has received scant research attention in the past. This demonstrates the importance of statutory planning as well as informal support to families to avoid added problems in child development and family bonding over and above that brought by the intellectual disabilities associated with Down syndrome, and the carer burden that families cope with.

Key words: Down syndrome, intellectual disabilities, births, incidence, deaths, hospitalisations

For peer review only - http://bmjopen.bmj.com/site/about/guidelines.xhtml

Strengths and limitations of this study

- A whole country population-based study on Down syndrome, with comprehensive identification via all NHS Scottish Regional Genetics Centres.
- 25 year period of linked data.
- Account taken of the potential confounders of age, gender, and extent of neighbourhood deprivation; underlying conditions were not investigated.
- Some of the Down syndrome individuals could not be matched, mostly due to missing data on post-code, and so were omitted from analyses.
- The use of routinely collected hospital and death statistics has many advantages including large scale coverage, but there is likely to be a degree of coding inaccuracy which is not quantified.

For peer review only

Introduction

Improvements in health and social care and attitudes to disability has increased the survival of people with Down syndrome in recent decades.[1-6] In England and Wales, it was estimated that there were 37,090 people with Down syndrome in 2011,[7] but exact numbers are not reported. The National Down Syndrome Cytogenetic Register for England and Wales produces data on the estimated live-birth rate for Down syndrome, using assumptions on termination rates for those receiving a pre-natal diagnosis. In 2013, it reported an estimated live birth rate of 1.0/1,000.[8] In the U.S.A., de Graaf et al[9] estimated live birth and population prevalence for Down syndrome for all 9 states in which data was available. They reported a live birth prevalence rate of 12.1/10,000, and an increase in the number of people living with Down syndrome from 1950 until 2010. In Canada, the live birth rate for Down syndrome was reported to have remained stable between 2005 and 2013, and higher than in the UK and USA rates, at approximately 13.5/10,000.[10] Similar data is not available for Scotland. Given the changing population demographic for people with Down syndrome, and limited evidence base, we require a better understanding of their live-birth rate and future health service needs.[4]

Down syndrome is associated with a range of congenital anomalies, particularly cardiac, and a higher risk for respiratory, immunological, endocrine, and gastrointestinal conditions.[4,11] This phenotype puts people with Down syndrome at risk of hospitalisation. This may impact on their development and family relationships, and so is an added disadvantage on top of their intellectual disabilities. Despite this, few previous studies have investigated hospitalisation of children and young people with Down syndrome, compared with the general population. Fitzgerald et al[12] studied hospital admissions of 405 children with Down

For peer review only - http://bmjopen.bmj.com/site/about/guidelines.xhtml

syndrome born in Western Australia between 1983-2004. They found the children with Down syndrome were hospitalised five times more often when compared with previously published general population data for the single year of 1995. They had no general population comparison data for length of stay. Zhu et al[13] investigated hospitalisations of 3,212 children and adults with Down syndrome compared to 67,204 children and adults without Down syndrome in Denmark, between 1977-2008. The people with Down syndrome had more than twice the rate of hospital admissions, and nearly three times as many bed days. Both studies found that length of admissions of the people with Down syndrome had reduced over the periods studied, and Zhu et al[13] reported more bed days for males, children under 5 years, and those with congenital heart anomalies. Other studies have lacked general population comparison data or been limited to single conditions. We are not aware of any large-scale studies specifically of children and young people with Down syndrome investigating these issues compared with their peers. There has been very little other investigation of the factors that influence length of hospital stay of children and young people with Down syndrome, and we located no studies on risk of readmission or on emergency admissions of children and young people.

Without accurate information on live-birth and death rates, and hospital admission needs, it is not possible to plan for the health care support that children with Down syndrome and their families need.

This study's aims were to investigate the (1) incidence of live births of people with Down syndrome over a 25 year period, (2) frequency of deaths of children and young people with Down syndrome compared to matched controls, and (3) hospital admission frequency and

For peer review only - http://bmjopen.bmj.com/site/about/guidelines.xhtml

duration, emergency admissions, and readmissions of children and young people with Down syndrome compared to matched controls.

Methods

Ethical approval

Permission to access, link and analyse these data was granted by the Privacy Advisory Committee (PAC) to the National Health Service (NHS) National Services Scotland (NSS) and the Registrar General. Written informed consent was not obtained from participants for their clinical records to be used in this study, but patient records were anonymized and de-identified prior to analysis. Safehaven approval was granted to approved researchers to analyse the data.

Patient and Public Involvement

This study was undertaken by the Scottish Learning Disabilities Observatory, which has a specific remit for people with intellectual disabilities, including people with Down syndrome; its steering group includes partners from third sector organisations, including Down Syndrome Scotland. Results from this study will be disseminated for people with Down syndrome and their families/carers in easy-read version via the Scottish Learning Disabilities Observatory website and newsletters.

Study sample, setting, and process

All NHS Scottish Regional Genetics Centres (the East, North, South-East and West Scotland Centres) identified all live-birth infants screened positive for Down syndrome postnatally (live births with trisomy 21, mosaic trisomy 21, or unbalanced translocation resulting in

For peer review only - http://bmjopen.bmj.com/site/about/guidelines.xhtml

trisomy 21) between 01st January 1990 to 01st December 2015. In Scotland, everyone is given a unique Community Health Index (CHI) at birth which is used in their health records. The CHI database is held centrally by National Services Scotland (NSS), and was used to identify five controls without Down syndrome for each person with Down syndrome, matched on sex, age (by month and year of birth), and neighbourhood deprivation (using the Scottish Index of Multiple Deprivation: SIMD).[14] Down syndrome infants who could not be matched due to missing data were excluded from analyses other than birth rate. The CHI also provides a means to record link each person identified with Down syndrome and their matched controls to routinely collected hospital admissions data (Scottish Morbidity Records 01: SMR01),[15] and National Records of Scotland death certificate data.[16] The end of the period of follow-up was 1st December 2015.

Data sources and definitions

Live-births: Live births of babies with Down syndrome from all NHS Scottish Regional Genetics Centres, 1990-2015; Scottish live births in the whole population, from the National Records of Scotland births time series, 1990-2015 (NRS, 2019).[17]

Death: Deaths and causes of death by the International Classification of Diseases codes[18] according to death certificates registered at National Records of Scotland.

Hospital admissions: SMR01 contains episode-based records for all non-psychiatric, non-obstetric acute hospital admissions in Scotland. The information collected includes the date of admission to hospital and discharge from it and whether the admission was routine or emergency. In SMR01, continuous periods of care are accounted for with a Continuous Inpatient Stay (CIS) marker. This CIS marker ensures a series of individual episodes over an unbroken period of care (e.g. transfers between wards or hospitals) can be identified and treated as one admission rather than several admissions. Transfer of a baby to a neonatal

For peer review only - http://bmjopen.bmj.com/site/about/guidelines.xhtml

intensive care unit after birth is counted as a first admission. Data Quality Assurance Assessments for SMR01 are performed periodically.[19,20]

Admission type: Admission type is coded as emergency or routine. "Emergency" admissions are those which were unplanned in advance; planned admissions are "routine" admissions.

Birth year group: Grouped into the most recent cohort (2004-2015) and the least recent cohort (1990-2015).

SIMD: SIMD matching with controls and reporting is by quintiles, where SIMD 1 is the most deprived neighbourhoods, and SIMD 5 is the most affluent neighbourhoods. SIMD is calculated at datazone level, identified from post codes.

Discharge type: Discharge type is coded as a regular discharge or irregular discharge. Irregular discharges include e.g. a patient discharging himself/herself against medical advice, or death.

All follow up/censoring: Children were followed up from birth, and all models were censored on death or 1st December 2015 (whichever came first) unless stated otherwise.

Analyses

Incidence of Down syndrome: Incidence was calculated for each calendar year from 1990 to 2015 inclusive.

Death: The mortality rates per 1,000 person years were described, split by age group [0-1 month of life, from 1 - 12 months, from 13 - 60 months, from 61 - 120 months, from 121 - 180 months and from 181 - 240 months], as were underlying causes of death, for people with Down syndrome and their matched controls.

First hospital admissions: First admissions for people with Down syndrome compared to controls were described, including by gender, SIMD, emergency admission, year of birth, and duration of first admissions in days. Descriptive data was compared using dependent t tests

For peer review only - http://bmjopen.bmj.com/site/about/guidelines.xhtml

for continuous variables and chi square tests for categorical variables or mann-whitney U-tests for length of hospital stay. The relative risk of first hospital admission was compared for people with Down syndrome and controls using Stratified (by sex, birth year group, SIMD) Cox regression models (log-log plots confirmed the proportional hazards assumption was met). The follow up period was defined as from date of birth until date of first admission, and admission type was entered as a potential confounder.

Risk of emergency hospital admission: The relative risk of emergency first hospital admission was compared for people with Down syndrome and controls, analysed using Stratified (by sex, birth year group, SIMD) Cox regression models. The follow up period was defined as from date of birth until date of first admission**.**

Duration of hospital admission: Conditional (stratified by sex, birth year group, SIMD) linear regression was used to model the duration of first hospital admission (log transformed to help negate potential skewing of results) as the outcome, comparing Down syndrome and control groups. Admission type was entered as a potential confounder.

Risk of re-admission: The relative risk of readmission (the next admission post the first admission) was compared for people with Down syndrome and controls, analysed using Stratified (by sex, birth year group, SIMD) Cox regression models. The follow up period was defined as date of discharge from the first admission until date of the next admission. First admission type, and discharge type from first admission were entered as potential confounders, and the interaction of group(Down syndrome versus controls)*admission type(emergency versus routine) was included.

Reference groups for all regression analyses were: not having Down syndrome, females, most affluent neighbourhoods, routine admissions, and regular type of hospital discharges. All analyses were conducted with IBM SPSS version 22.

For peer review only

For peer review only - http://bmjopen.bmj.com/site/about/guidelines.xhtml

Results

Between 01st January 1990 to 01st December 2015, the Scottish Regional Genetics Centres identified 1,479 live-births with Down syndrome. 1,291 were matched with 6,455 controls. 188 (12.7%) infants with Down syndrome were excluded from all analyses except birth incidence, as they could not be matched due to missing data on SIMD quintile (n=160) in view of missing post-code information, sex (n=21), month and year of birth (n=5), and CHI e.g. due to migration out of Scotland (n=2). Of the 1,479 infants born with Down syndrome, 689 (46.6%) were girls, 769 (51.9%) were boys, and sex was not recorded for 21 (1.4%).The proportion of births for the Down syndrome population, by the 5 SIMD quintiles were: 1= 21.3%, 2= 20.4%, 3=18.5%, 4= 20.1% and 5=19.7%, and SIMD data was missing for 160 (10.8%). Of the 1,291 infants with Down syndrome with matched controls, 613 (47.5%) were girls, 678 (52.5%) were boys. The proportion of births for the Down syndrome population, by the 5 SIMD quintiles were: 1= 21.2%, 2= 20.5%, 3=18.3%, 4= 20.1% and 5=19.8%. Hence they appear to be representative of all the Down syndrome infants that were born.

Incidence of Down syndrome live-births over time

Given the total number of infants born was 1,508,483, the incidence of Down syndrome live-births was 1,479/1,508,483 (1.0/1,000 births). Table 1 and figure 1 show the incidence of Down syndrome live-births for each year of the study period (with a moving average smoothed trend line to account for noise from year on year variation). The birth rate in Scotland has fallen since the early 1990s, whilst the incidence of Down syndrome shows some year-to-year variation and appears to have remained the same or risen since the early 1990s.

For peer review only - http://bmjopen.bmj.com/site/about/guidelines.xhtml

Table 1: Incidence of Down syndrome by year of birth

Year of birth	Number of births registered in Scotland (all people)	Down Syndrome	
	N	N	(%)
1990	65,973	60	0.09%
1991	67,024	66	0.10%
1992	65,789	38	0.06%
1993	63,337	35	0.06%
1994	61,656	43	0.07%
1995	60,051	46	0.08%
1996	59,296	40	0.07%
1997	59,440	72	0.12%
1998	57,319	54	0.09%
1999	55,147	52	0.09%
2000	53,076	60	0.11%
2001	52,527	61	0.12%
2002	51,270	44	0.09%
2003	52,432	58	0.11%
2004	53,957	79	0.15%
2005	54,386	63	0.12%
2006	55,690	44	0.08%
2007	57,781	70	0.12%
2008	60,041	63	0.10%
2009	59,046	73	0.12%
2010	58,791	59	0.10%
2011	58,590	63	0.11%
2012	58,027	59	0.10%
2013	56,014	58	0.10%
2014	56,725	62	0.11%
2015	55,098	57	0.11%*
**Total**	**1,508,483**	**1,479**	**0.10%**

*In 2015 the Down syndrome total is for 11 months only, so the % in 2015 is adjusted to 11/12 months in the general population

Deaths

Of the 1,291 people with Down syndrome with matched controls, 92 (7.1%) died during the study period (13,840 person years of follow up for the Down syndrome group), of whom 47

For peer review only - http://bmjopen.bmj.com/site/about/guidelines.xhtml

(51.0%) were female, 45 (48.9%) were male. Of the 6,455 matched controls, 23 (0.4%) died during the period (74,778 person years follow up for the controls), of whom 9 (39.1%) were females, and 14 (60.9%) were males. Death was therefore 18 times more common in the Down syndrome group. Most of the deaths of the people with Down syndrome occurred in infancy. Death rates per 1,000 of the population by age groups for the Down syndrome population versus controls were; 8.5 (n=11) versus 0 for the 1st month of life, 45.1 (n=53) versus 1.95 (n=12) from 1 - 12 months, 19.4 (n=18) versus 1.21 (n=6) from 13 - 60 months, 10.8 (n=7) versus 0.85 (<5) from 61 - 120 months, 7.59 (n=<5) versus 0 from 121 - 180 months and 0 versus 1.82 (<5) from 181 - 240 months. The most common certified underlying causes of death for the Down syndrome group were congenital heart anomalies (n=33, 34.4%), Down syndrome (n=26, 27.1%) and infection (excludes respiratory infections) (n=11, 11.5%). This pattern was different compared to controls in whom causes of deaths were due to a range of other factors not found for people with Down syndrome (n=8, 34.8%), prematurity/perinatal causes (n=5, 21.7%) and congenital heart anomalies (n=5, 21.7%). Although less common than some other causes, leukaemia was a cause of death among people with Down syndrome (n=<5) compared to controls where no deaths were from leukaemia.

Admissions

Table 2 presents details on the first all-cause hospital admissions for the two groups. 1,130/1,291 (87.5%) of the Down syndrome group had at least one hospital admission compared to 3,351/6,455 (51.9%) of the control group (χ^2=559.664; df=1; P<0.001). Compared with the control group, the Down syndrome group were statistically younger at the time of first hospital admission (t(4500)=16.0, P<.001), had less admissions among females (χ^2=559.664; df=1; P<0.001), were more equally spread across neighbourhoods whilst the

For peer review only - http://bmjopen.bmj.com/site/about/guidelines.xhtml

control group had a gradient across SIMD groups with those in the most deprived areas having more hospital admissions (χ^2=567.088; df=1; P<0.001), and had longer duration of admissions (5.03 days [Median=2], versus 1.78 days [Median=1]) by 3.25 days (U=1296817, P<0.001). The rate of admissions slightly decreased over time for people with Down syndrome (from 528, 90.7% [in 1990-2003] to 602, 84.9% [in 2004-2015]) and decreased for controls (from 1,837, 63.3% [in 1990-2003] to 1,514, 43.0% [in 2004-2015]; t(22294)=-3.146, P<0.001). The gap between the higher rate of admissions has widened over time for people with Down syndrome compared to controls.

Table 2: First all-cause hospital admissions

		**Down syndrome (n=1,130)**	**Control Group (n=3,351)**	**P-value+**
		Mean(SD), N(%) Median(IQR)	Mean(SD), N(%), Median(IQR)	
Age at admission (years)		1.06 (SD=2.4)	3.33 (SD=4.6)	<0.001
Sex	Male	586 (51.9%)	1,873 (55.9%)	<0.001
	Female	544 (48.1%)	1,478 (44.1%)	
Birth year group	Most recent	602 (84.9%)	1,514 (43.0%)	<0.001
	Less recent	528 (90.7%)	1,837 (63.3%)	
SIMD	Most affluent (5)	218 (19.3%)	575 (17.2%)	<0.001
	4	231 (20.4%)	619 (18.5%)	
	3	208 (18.4%)	602 (18.0%)	
	2	237 (21.0%)	732 (21.9%)	
	Most deprived (1)	236 (20.9%)	822 (24.5%)	
Admission type	Emergency	882 (78.1%)	2,401 (71.6%)	N/S
	Routine	248 (21.9%)	950 (28.3%)	
Duration of admission (days)		2.00 (IQR=1.00-4.00)	1.00 (IQR=1.00-1.00)	<0.001

Incl=including; SD=Standard deviation; IQR=Interquartile Range; SIMD=Scottish Index of Multiple Deprivation
Denominator for birth year group most recent/less recent are for Down syndrome n= 709/582/Control group n=3,555/2,900.

For peer review only - http://bmjopen.bmj.com/site/about/guidelines.xhtml

Table 3 displays the results of the Cox regressions for time to first all-cause hospital admission. The risk of admission was higher amongst people with Down syndrome compared to controls, with an unadjusted hazard ratio (HR)=2.74 (95% CI 2.52-2.98). This remained after adjusting for type of first admission: HR=1.84 (95% CI 1.68-2.01).

Table 3: Risk for first all cause hospital admission

		Separately entered		Simultaneously entered	
		**HR value**	**95% CI**	**HR value**	**95% CI**
Group	No Down syndrome	ref	ref	ref	ref
	Down syndrome	2.74**	2.52-2.98	1.84**	1.68-2.01
First admission type	Routine	ref	ref	ref	ref
	Emergency	1.10**	1.00-1.20	1.19**	1.08-1.30

*P<.05; **P<.001
HR=Hazard Ratio; CI=Confidence Interval; SIMD=Scottish Index of Multiple Deprivation

Table 4 displays the results of the Cox regressions for time to first emergency hospital admission. The risk of emergency admission was higher amongst people with Down syndrome compared to controls, with a hazard ratio (HR)=2.87 (95% CI 2.61-3.15).

Table 4: Risk for first emergency hospital admission

		Separately entered	
		**HR value**	**95% CI**
Group	No Down syndrome	ref	ref
	Down syndrome	2.87**	2.61-3.15

*P<.05; **P<.001
HR=Hazard Ratio; CI=Confidence Interval

For peer review only - http://bmjopen.bmj.com/site/about/guidelines.xhtml

Duration of hospitalisation

The duration of first hospital admission was significantly longer for the Down syndrome group at 5.03 days [Median=2 days] versus 1.78 days [Median=1 day]) by 3.25 days. After adjustment (with a log transformed time variable, to help account for any data skew) for sex, birth year group, SIMD, and type of admission, the Coefficient=0.25 (CI 0.22-0.28) (table 5). This log transformed Coefficient can be interpreted as a ratio of difference for the Down syndrome population compared to controls (exponentiate of the log transformed coefficient, exp(0.248)=1.28145993219 – 1 x 100=28). This means the average length of stay increased by 28% for being in the Down syndrome population, compared to the controls.

Table 5: Duration of first all-cause hospital admission (log transformed time variable)

		**Separately entered**		**Simultaneously entered**	
		**Coef**	**95% CI**	**Coef**	**95% CI**
Group	No Down syndrome	ref	ref	ref	ref
	Down syndrome	0.24**	0.22-0.27	0.25**	0.22-0.28
First admission type	Routine	ref	ref	ref	ref
	Emergency	0.02	0.00-0.04	0.04**	0.02-0.06

*P<.05; **P<.001
Coef=Coefficient; CI=Confidence Interval

Risk of re-admission

Of the Down syndrome group, 963/1,130 (85.2%) had at least one re-admission, compared to 1,700/3,351 (50.7%) of the control group (χ^2=132.299; df=1; P<0.001). The baseline age at readmission was lower for the Down syndrome group with a mean age of 1.16 years (SD=1.79) compared to the controls with a mean age of 1.44 years (SD=2.25; t(7772)=5.16, P<.001).The unadjusted HR of Down syndrome for time to readmission following discharge from first admission was 2.42 (95% CI 2.12-2.77). The HR when adjusted for type of admission, type of first discharge and group*emergency interaction was 2.55 (95% CI 2.08-3.14). Results are displayed in table 6.

For peer review only - http://bmjopen.bmj.com/site/about/guidelines.xhtml

Table 6: Time to readmission following discharge from first admission

		Separately entered		Simultaneously entered	
		**HR value**	**95% CI**	**HR value**	**95% CI**
Group	No Down syndrome	ref	ref	ref	ref
	Down syndrome	2.42**	2.12-2.77	2.55**	2.08-3.14
First admission type	Routine	ref	ref	ref	ref
	Emergency	0.97*	0.83-1.13	1.39	1.05-1.84
Discharge type	Routine	ref	ref	ref	ref
	Irregular	0.86	0.83-0.89	0.85	0.82-0.88
Group*first admission type	-	-	-	1.07	0.78-1.45

*P<.05; **P<.001

HR=Hazard Ratio; CI=Confidence Interval

For peer review only

Discussion

Principal findings and interpretation

We found the Scottish incidence of Down syndrome live-births to be 1.0/1,000 births over the last 25 years, with it being possibly higher now than in the early 1990s. The Scottish birth rate has fallen overall, but incidence of Down syndrome has not. Death rate was 18 times higher for the children/young people with Down syndrome than for their controls, and their causes of death differed, including causes that might be expected to have been amenable to good health care such as infections.

Over a 25 year period, we found that children/young people with Down syndrome compared with matched controls were considerably more likely to have had at least one hospital admission (87.5% compared to 51.9%; adjusted HR=1.84), and re-admissions (85.2% compared to 50.7%; adjusted HR=2.55). Their admissions were more likely to have been emergency admissions (78.1% compared to 71.6%; first emergency admission HR=2.87), and of longer duration (5.03 days compared to 1.78 days; adjusted (log transformed Coef=.25). Given the higher rate of first emergency admissions in the Down syndrome group, we included the interaction of group x type of first admission (emergency or routine) in the

analysis of readmissions, but they still remained more likely to be readmitted. We are not aware of any previous study of readmissions and emergency admissions in this population. The rate of admissions only slightly decreased over time (from 1990-2003 to 2004-2015) for the children/young people with Down syndrome (90.7% to 84.9%), whilst it decreased to a much larger extent for the control group (from 63.3% to 43.0%), perhaps reflecting changes in medical practice over time, to keep children out of hospital as far as possible, but admitting for serious problems which the Down syndrome children may be more likely to experience. These findings are important, as they demonstrate considerable extra burden to both children and young people with Down syndrome, and to their families. This demonstrates the importance of statutory as well as informal support to families to avoid added problems in child development over and above the intellectual disabilities that the children with Down syndrome experience, and the carer burden that families cope with. Additionally, the high rates of emergency admissions and re-admissions may indicate some poorer quality management of health care at the primary care level (to avoid admissions), and have not to our knowledge been reported before, though other factors, including underlying health conditions may contribute.

The Down syndrome group were younger at the time of first hospital admission, had a more equal sex distribution for first admission than the control group, and an equal distribution across extent of neighbourhood deprivation, whilst the control group had a gradient with those in the most deprived areas more likely to have had a hospital admission. This suggests that at present, the children with Down syndrome appear to receive comparable care in terms of first admission, regardless of their neighbourhood, and highlights that findings in the general population cannot be relied upon when planning services for people with Down syndrome/intellectual disabilities, such as focussing services in areas of greatest deprivation.

For peer review only - http://bmjopen.bmj.com/site/about/guidelines.xhtml

Families of children and young people with Down syndrome need focused support regardless of the areas they reside in.

Comparison with previous literature

As well as being 18 times more likely to die than their controls, we found that the children/young people with Down syndrome were more likely to be hospitalised, emergency hospitalised, re-admitted, and for longer durations. We have not identified any previous studies on re-admission with which to compare our findings, nor on emergency admissions. For the earlier period of 1977-2008 in Denmark, Zhu et al[13] reported that children and adults with Down syndrome had more than twice the rate of hospital admissions (all admissions, not separately reported for first admissions or repeat admissions), similar to our adjusted hazard ratios for first and repeat admissions, but we specifically investigated children and young people unlike that study. The smaller Australian study for the earlier period of 1983-2004 suggested the hospitalisation rate was five times higher, but relied on previously reported general population data from 1995.[12]

As our study shows, and as previously reported in a study of children with Down syndrome identified from population-based sources in 1997 (n=210) and 2004 (n=208) in Western Australia,[21] it is highly likely that admission rates change over time, so these two studies are not strictly comparable to ours, and we have found no studies contemporaneous with ours with which to draw comparisons. Previous, small scale studies reported length of admissions of children with Down syndrome to be 4.7 days in a Swedish study of 211 children followed up for at least 17 years,[22] and 7 days specifically for bronchiolitis in a small retrospective case-control study in Singapore,[23] compared with the 5.0 days we found. A further small

For peer review only - http://bmjopen.bmj.com/site/about/guidelines.xhtml

study of 213 children with Down syndrome aged 0-3 years in America reported high admission rates.[24]

The live birth incidence for Down syndrome we report is similar to that recently reported in England, USA, and Canada.[8-10] Antenatal screening changes may underlie the variability in the birth rates found over time. Previous research in Scotland showed that decisions to terminate pregnancy following antenatal diagnosis of aneuploidy had fallen over time,[1] which our results are also possibly suggestive of from the early 1990s to more recently. In contrast, in some other countries (e.g. Denmark), following the introduction of antenatal combined screening for Down syndrome in 2004, there was a concurrent increase in prenatal diagnoses and the number of Down syndrome live births decreased suddenly and significantly before then stabilising.[25] A higher proportion of the Scottish population have catholic faith compared with Denmark which might contribute in part to differences between countries: a range of complex factors influence women's decision-making regarding prenatal screening and diagnosis of Down syndrome, and it is possible that more societal acceptance of diversity and people with disabilities, or better availability of family support may influence decisions in Scotland over time,[1,26] although this requires further investigation.

Strengths and limitations

This is the first population-based study examining incidence of Down syndrome and health care utilisation over time in Scotland compared to the general population, and the first worldwide to investigate emergency hospital admissions and readmissions. It controlled for the potential confounders such of age, sex, and SIMD. It was not subject to selection bias, as the population identification was based on routine data sources from all genetic service laboratories in Scotland (i.e. the whole population). The cox regression analysis for risk of

For peer review only - http://bmjopen.bmj.com/site/about/guidelines.xhtml

admissions and readmissions provides advantages over logistic or linear techniques as this includes consideration of time to event (person time at risk). However, there was some missing control data, mostly due to missing post-code data, and this is a limitation of the study. There was no data on ethnicity which is a limitation; the Scottish population is 96% Caucasian. The use of routinely collected hospital and death statistics has many advantages including large scale coverage, but there is likely to be a degree of coding inaccuracy which is not quantified. We did not aim to investigate underlying conditions which are associated with admissions and death in the two groups.

Conclusions

As people with Down syndrome are living longer than in the past, we have provided contemporaneous statistics for the live birth rate of babies with Down syndrome and their childhood death rate compared with peers. We have found that their increasing survival is not without health burden for the child, their family, and health services – we report the majority require hospital admissions, readmissions, and longer lengths of stays than their peers, which has received scant research attention in the past. Some factors may even suggest poorer health care than their peers, such as higher rates of emergency admissions, although we did not aim to investigate the conditions associated with admission. Together this brings important new findings necessary to address the challenge to plan support that children with Down syndrome and their families need. They need adequate health care, and also an awareness of the impact that hospitalisation can have on child development and family bonding. There may be a need to develop tailored packages of support for families with children/young people with Down syndrome to cope with the higher burden of health care needs of their offspring.

For peer review only - http://bmjopen.bmj.com/site/about/guidelines.xhtml

Footnotes

Contributors: LAH-M contributed to the study design, the analysis and interpretation of data, drafted the first article and revised it critically; DM, AH, LO contributed to study design, interpretation of data and revised the article critically; S-AC, JP, and RM conceived and designed the study, contributed to study analysis, interpretation of data and revised the article critically. All authors approved the final version

Funding: MRC Mental Health Data Pathfinder Award (MC_PC_17217), and the Scottish Government via the Scottish Learning Disabilities Observatory

Competing interests: The authors declare no competing interests

Ethical approval: Approval was gained from the Privacy Advisory Committee (PAC)

Word count: 4553

Data sharing statement: All data relevant to the study are included in the article or uploaded as supplementary information.

For peer review only

For peer review only - http://bmjopen.bmj.com/site/about/guidelines.xhtml

Reference

1. Jacobs M, Cooper S-A., McGowan R, Nelson S, Pell J. Pregnancy outcome following prenatal diagnosis of chromosomal anomaly: a record linkage study of 26,261 pregnancies. *PLoS ONE,* 2016; 11(12), e0166909.
2. Carothers AD, Boyd E, Lowther G, et al. Trends in prenatal diagnosis of Down syndrome and other autosomal trisomies in Scotland 1990 to 1994, with associated cytogenetic and epidemiological findings. *Genet Epidemiol.* 1999;16(2):179-90.
3. O'Leary L, Cooper SA, Hughes-McCormack L. Early death and causes of death of people with intellectual disabilities: A systematic review. *J Appl Res Intellect Disabil.* 2018;31(3):325-42.
4. Jensen KM, Bulova PD. Managing the care of adults with Down's syndrome. *BMJ.* 2014;349:g5596.
5. Hijii T, Fukushige J, Igarashi H, Takahashi N, Ueda K. Life expectancy and social adaptation in individuals with Down syndrome with and without surgery for congenital heart disease. *Clin Pediatr (Phila).* 1997;36(6):327-32.
6. Bull C, Rigby ML, Shinebourne EA. Should management of complete atrioventricular canal defect be influenced by coexistent Down syndrome. *Lancet.* 1985;1(8438):1147-9.
7. Wu J, Morris JK. The population prevalence of Down's syndrome in England and Wales in 2011. *Eur J Hum Genet.*2013;21(9):1016-9.
8. Morris, J.K., and Springett, A. (2014). The National Down Syndrome Cytogenetic Register for England and Wales: 2013 Annual Report. National Down Syndrome Cytogenetic Register. Queen Mary University of London.
9. de Graaf G, Buckley F, Skotko BG. Estimates of the live births, natural losses, and elective terminations with Down syndrome in the United States. *Am J Med Genet A.* 2015;167A(4):756-67.
10. Public Health Agency of Canada. Down syndrome surveillance in Canada, 2005-2013. Ottawa, 2017.
11. Ram G, Chinen J. Infections and immunodeficiency in Down syndrome. *Clin Exp Immunol.* 2011;164(1):9-16.
12. Fitzgerald P, Leonard H, Pikora TJ, Bourke J, Hammond G. Hospital admissions in children with down syndrome: experience of a population-based cohort followed from birth. *PLoS ONE.* 2013;8(8):e70401.
13. Zhu JL, Hasle H, Correa A, et al. Hospitalizations among people with Down syndrome: a nationwide population-based study in Denmark. *Am J Med Genet A.* 2013;161A(4):650-7.
14. National Statistics. Scottish Index of Multiple Deprivation 2012. Edinburgh: The Scottish Government; 2012.
15. Scottish Government, 2016: http://www.gov.scot/Topics/Statistics/SIMD)
16. Scottish Morbidity Records 01. http://www.ndc.scot.nhs.uk/Data-Dictionary/SMR-Datasets//SMR01-General-Acute-Inpatient-and-Day-Case/)
17. National Records of Scotland, (2019): https://www.nrscotland.gov.uk/statistics-and-data/statistics/statistics-by-theme/vital-events/births/births-time-series-data).
18. World Health Organisation.International Statistical Classification of Diseases and Related Health Problems, 10th Revision. Geneva: World Health Organisation, 1992.
19. Information Services Division (ISD) Scotland: Assessment of SMR01 Data 2010-2011. Scotland Report; May 2012
20. Turner M, Barber M, Dodds H, Dennis M, Langhorne P, Macleod MJ. Agreement between routine electronic hospital discharge and Scottish Stroke Care Audit (SSCA)

For peer review only - http://bmjopen.bmj.com/site/about/guidelines.xhtml

data in identifying stroke in the Scottish population. *BMC Health Serv Res*. 2015 Dec 30;15:583.
21. Thomas K, Bourke J, Girdler S, et al. Variation over time in medical conditions and health service utilization of children with Down syndrome. *J Pediatr*. 2011;158:194–200
22. Frid C, Anneren G, Rasmussen F, Sundelin C, Drott P. Utilization of medical care among children with Down's syndrome. *J Intellect Disabil Res*. 2002;46:310–317
23. Ting TW, Chan HY, Wong PPC, Testoni D, & Lee JH. Down syndrome increases hospital length of stay in children with bronchiolitis. *Proceedings of Singapore Healthcare*. 2016; *25*(1), 64–67.
24. So SA, Urbano RC, Hodapp RM. Hospitalizations of infants and young children with Down syndrome: evidence from inpatient person-records from a statewide administrative database. *J Intellect Disabil Res.* 2007;51(Pt 12):1030-8.
25. Lou, S, Petersen, OB, Jørgensen, FS, Lund, ICB, Kjærgaard, S, Danish Cytogenetic Central Registry Study Group, et al. National screening guidelines and developments in prenatal diagnoses and live births of Down syndrome in 1973–2016 in Denmark. *Acta Obstet Gynecol Scand* 2018; 97: 195– 203.
26. Olsen CL, Cross PK, Gensburg LJ. Down syndrome: interaction between culture, demography, and biology in determining the prevalence of a genetic trait. *Hum Biol.* 2003; 75(4):503-20.

For peer review only - http://bmjopen.bmj.com/site/about/guidelines.xhtml

Figure 1: Incidence of Down syndrome births by year in Scotland from 1990-2015

For peer review only

For peer review only - http://bmjopen.bmj.com/site/about/guidelines.xhtml

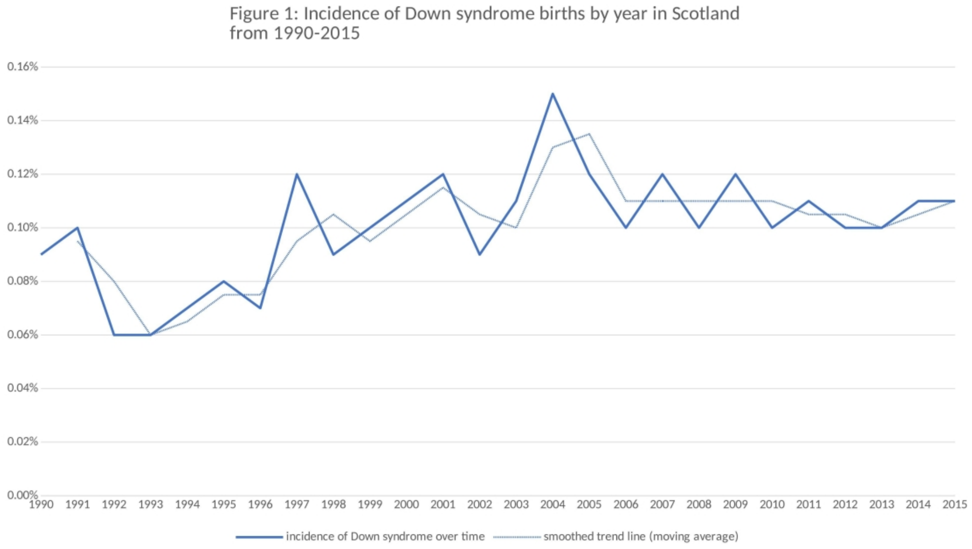

Figure 1: Incidence of Down syndrome births by year in Scotland from 1990-2015

338x190mm (300 x 300 DPI)

STROBE Statement—Checklist of items that should be included in reports of ***cohort studies***

	Item No	Recommendation	Page No
**Title and abstract**	1	(*a*) Indicate the study's design with a commonly used term in the title or the abstract<br>(*b*) Provide in the abstract an informative and balanced summary of what was done and what was found	2
**Introduction**			
Background/rationale	2	Explain the scientific background and rationale for the investigation being reported	4/5
Objectives	3	State specific objectives, including any prespecified hypotheses	5
**Methods**			
Study design	4	Present key elements of study design early in the paper	6
Setting	5	Describe the setting, locations, and relevant dates, including periods of recruitment, exposure, follow-up, and data collection	6-7
Participants	6	(*a*) Give the eligibility criteria, and the sources and methods of selection of participants. Describe methods of follow-up<br>(*b*) For matched studies, give matching criteria and number of exposed and unexposed	6-8
Variables	7	Clearly define all outcomes, exposures, predictors, potential confounders, and effect modifiers. Give diagnostic criteria, if applicable	6-8
Data sources/ measurement	8*	For each variable of interest, give sources of data and details of methods of assessment (measurement). Describe comparability of assessment methods if there is more than one group	6-8
Bias	9	Describe any efforts to address potential sources of bias	8
Study size	10	Explain how the study size was arrived at	6
Quantitative variables	11	Explain how quantitative variables were handled in the analyses. If applicable, describe which groupings were chosen and why	7-8
Statistical methods	12	(*a*) Describe all statistical methods, including those used to control for confounding<br>(*b*) Describe any methods used to examine subgroups and interactions<br>(*c*) Explain how missing data were addressed<br>(*d*) If applicable, explain how loss to follow-up was addressed<br>(*e*) Describe any sensitivity analyses	7-8
**Results**			
Participants	13*	(a) Report numbers of individuals at each stage of study—eg numbers potentially eligible, examined for eligibility, confirmed eligible, included in the study, completing follow-up, and analysed<br>(b) Give reasons for non-participation at each stage<br>(c) Consider use of a flow diagram	9-10
Descriptive data	14*	(a) Give characteristics of study participants (eg demographic, clinical, social) and information on exposures and potential confounders<br>(b) Indicate number of participants with missing data for each variable of interest<br>(c) Summarise follow-up time (eg, average and total amount)	9-14
Outcome data	15*	Report numbers of outcome events or summary measures over time	11-15

For peer review only

Main results	16	(*a*) Give unadjusted estimates and, if applicable, confounder-adjusted estimates and their precision (eg, 95% confidence interval). Make clear which confounders were adjusted for and why they were included<br>(*b*) Report category boundaries when continuous variables were categorized<br>(*c*) If relevant, consider translating estimates of relative risk into absolute risk for a meaningful time period	11-15
Other analyses	17	Report other analyses done—eg analyses of subgroups and interactions, and sensitivity analyses	11-15
**Discussion**			
Key results	18	Summarise key results with reference to study objectives	15-16
Limitations	19	Discuss limitations of the study, taking into account sources of potential bias or imprecision. Discuss both direction and magnitude of any potential bias	18
Interpretation	20	Give a cautious overall interpretation of results considering objectives, limitations, multiplicity of analyses, results from similar studies, and other relevant evidence	18-19
Generalisability	21	Discuss the generalisability (external validity) of the study results	18-19
**Other information**			
Funding	22	Give the source of funding and the role of the funders for the present study and, if applicable, for the original study on which the present article is based	19

*Give information separately for exposed and unexposed groups.

Note: An Explanation and Elaboration article discusses each checklist item and gives methodological background and published examples of transparent reporting. The STROBE checklist is best used in conjunction with this article (freely available on the Web sites of PLoS Medicine at http://www.plosmedicine.org/, Annals of Internal Medicine at http://www.annals.org/, and Epidemiology at http://www.epidem.com/). Information on the STROBE Initiative is available at http://www.strobe-statement.org.

For peer review only

BMJ Open

Birth incidence, deaths, and hospitalisations of children and young people with Down syndrome, 1990-2015: birth cohort study

Journal:	*BMJ Open*
Manuscript ID	bmjopen-2019-033770.R2
Article Type:	Original research
Date Submitted by the Author:	03-Mar-2020
Complete List of Authors:	Hughes-McCormack, Laura; University of Glasgow, Institute of Health and Wellbeing<br>McGowan, Ruth; West of Scotland Regional Genetics Service<br>Pell, J. P.; Univ Glasgow<br>Mackay, Daniel; University of Glasgow<br>Henderson, Angela; University of Glasgow, Institute of Health and Wellbeing<br>O'Leary, Lisa; Edinburgh Napier University<br>Cooper, Sally-Ann; Glasgow University , Institute of Health and Wellbeing
**Primary Subject Heading**:	Health services research
Secondary Subject Heading:	Health services research, Epidemiology
Keywords:	Down syndrome, intellectual disabilities, births, incidence, deaths, hospitalisations

SCHOLARONE™
Manuscripts

I, the Submitting Author has the right to grant and does grant on behalf of all authors of the Work (as defined in the below author licence), an exclusive licence and/or a non-exclusive licence for contributions from authors who are: i) UK Crown employees; ii) where BMJ has agreed a CC-BY licence shall apply, and/or iii) in accordance with the terms applicable for US Federal Government officers or employees acting as part of their official duties; on a worldwide, perpetual, irrevocable, royalty-free basis to BMJ Publishing Group Ltd ("BMJ") its licensees and where the relevant Journal is co-owned by BMJ to the co-owners of the Journal, to publish the Work in this journal and any other BMJ products and to exploit all rights, as set out in our licence.

The Submitting Author accepts and understands that any supply made under these terms is made by BMJ to the Submitting Author unless you are acting as an employee on behalf of your employer or a postgraduate student of an affiliated institution which is paying any applicable article publishing charge ("APC") for Open Access articles. Where the Submitting Author wishes to make the Work available on an Open Access basis (and intends to pay the relevant APC), the terms of reuse of such Open Access shall be governed by a Creative Commons licence – details of these licences and which Creative Commons licence will apply to this Work are set out in our licence referred to above.

Other than as permitted in any relevant BMJ Author's Self Archiving Policies, I confirm this Work has not been accepted for publication elsewhere, is not being considered for publication elsewhere and does not duplicate material already published. I confirm all authors consent to publication of this Work and authorise the granting of this licence.

For peer review only

Birth incidence, deaths, and hospitalisations of children and young people with Down syndrome, 1990-2015: birth cohort study

Laura Anne Hughes-McCormack[1]

Ruth McGowan[2]

Jill P. Pell[3]

Daniel Mackay[3]

Angela Henderson[1]

Lisa O'Leary[4]

*Sally-Ann Cooper[1]

***Correspondence:**

Institute of Health and Wellbeing, University of Glasgow

Mental Health and Wellbeing group, Administrative Building, Gartnavel Royal Hospital,

1055 Great Western Road, Glasgow, G12 0XH, UK.

Sally-Ann.Cooper@glasgow.ac.uk

1. Mental Health and Wellbeing research group, Institute of Health and Wellbeing, University of Glasgow, Glasgow, UK
2. West of Scotland Regional Genetics Service, Queen Elizabeth University Hospital, Glasgow, UK
3. Public Health, Institute of Health and Wellbeing, University of Glasgow, Glasgow, UK
4. School of Health and Social Care, Edinburgh Napier University, Edinburgh, UK

Key words: Down syndrome, intellectual disabilities, births, incidence, deaths, hospitalisations

Word count: 4973

For peer review only - http://bmjopen.bmj.com/site/about/guidelines.xhtml

Abstract

Objective: To investigate current Down syndrome live birth and death rates, and childhood hospitalisations, compared with peers.

Setting: General community.

Participants: All live-births with Down syndrome, 1990-2015, identified via Scottish regional cytogenetic laboratories, each age-sex-neighbourhood deprivation matched with 5 non-Down syndrome controls. Record-linkage to Scotland's hospital admissions and death data.

Primary outcome: Hazard ratios comparing risk of first hospitalisation (any and emergency), readmission for children with Down syndrome and matched controls were calculated using stratified Cox PH hazards model, and length of hospital stay was calculated using a conditional log-linear regression model.

Results: 689/1,479 (46.6%) female and 769/1,479 (51.9%) male children/young people with Down syndrome were identified (1.0/1,000 births, with no reduction over time); 1,235 were matched. 92/1,235 (7.4%) died during the period, 18.5 times more than controls. More of the Down syndrome group had at least one admission (IRR 72.89 [68.72-77.32] versus 40.51 [39.15-41.92]; adjusted HR=1.84 [1.68, 2.01]), and re-admissions (IRR 54.85 [51.46-58.46] versus 15.06 [14.36-15.80]; adjusted HR=2.56 [2.08, 3.14]). More of their admissions were emergencies (IRR 56.78 [53.13-60.72] versus 28.88 [27.73-30.07]; first emergency admission adjusted HR=2.87 [2.61, 3.15]). Children with Down syndrome had 28% longer first admission after birth. Admission rate increased from 1990-2003 to 2004-2014 for the Down syndrome group (90.7% to 92.2%), and decreased for controls (63.3% to 44.8%).

Conclusions: We provide contemporaneous statistics on the live birth rate of babies with Down syndrome, and their childhood death rate. They require more hospital admissions, readmissions, and emergency admissions, and longer lengths of stays than their peers, which has received scant research attention in the past. This demonstrates the importance of statutory planning as well as informal support to families to avoid added problems in child development and family bonding over and above that brought by the intellectual disabilities associated with Down syndrome.

For peer review only

Strengths and limitations of this study

- A whole country population-based study on Down syndrome, with comprehensive identification via all NHS Scottish Regional Genetics Centres.
- 25 year period of linked data.
- Account taken of the potential confounders of age, sex and extent of neighbourhood deprivation; underlying conditions were not investigated.
- Some of the Down syndrome individuals could not be matched, mostly due to missing data on post-code, and so were omitted from analyses.
- The use of routinely collected hospital and death statistics has many advantages including large scale coverage, but there is likely to be a degree of coding inaccuracy which is not quantified.

For peer review only

For peer review only - http://bmjopen.bmj.com/site/about/guidelines.xhtml

Introduction

Improvements in health and social care and attitudes to disability has increased the survival of people with Down syndrome in recent decades.[1-6] In England and Wales, it was estimated that there were 37,090 people with Down syndrome in 2011,[7] but exact numbers are not reported. The National Down Syndrome Cytogenetic Register for England and Wales produces data on the estimated live-birth rate for Down syndrome, using assumptions on termination rates for those receiving a pre-natal diagnosis. In 2013, it reported an estimated live birth rate of 1.0/1,000.[8] In the U.S.A., de Graaf et al[9] estimated live birth and population prevalence for Down syndrome for all 9 states in which data was available. They reported a live birth prevalence rate of 12.1/10,000, and an increase in the number of people living with Down syndrome from 1950 until 2010. In Canada, the live birth rate for Down syndrome was reported to have remained stable between 2005 and 2013, and higher than in the UK and USA rates, at approximately 13.5/10,000.[10] Similar data is not available for Scotland. Given the changing population demographic for people with Down syndrome, and limited evidence base, we require a better understanding of their live-birth rate and future health service needs.[4]

Down syndrome is associated with a range of congenital anomalies, particularly cardiac, and a higher risk for respiratory, immunological, endocrine, and gastrointestinal conditions.[4,11] This phenotype puts people with Down syndrome at risk of hospitalisation. This may impact on their development and family relationships, and so is an added disadvantage on top of their intellectual disabilities. Despite this, few previous studies have investigated hospitalisation of children and young people with Down syndrome, compared with the general population. Fitzgerald et al[12] studied hospital admissions of 405 children with Down

For peer review only - http://bmjopen.bmj.com/site/about/guidelines.xhtml

syndrome born in Western Australia between 1983-2004. They found the children with Down syndrome were hospitalised five times more often when compared with previously published general population data for the single year of 1995. They had no general population comparison data for length of stay. Zhu et al[13] investigated hospitalisations of 3,212 children and adults with Down syndrome compared to 67,204 children and adults without Down syndrome in Denmark, between 1977-2008. The people with Down syndrome had more than twice the rate of hospital admissions, and nearly three times as many bed days. Both studies found that length of admissions of the people with Down syndrome had reduced over the periods studied, and Zhu et al[13] reported more bed days for males, children under 5 years, and those with congenital heart anomalies. Other studies have lacked general population comparison data or been limited to single conditions. We are not aware of any large-scale studies specifically of children and young people with Down syndrome investigating these issues compared with their peers. There has been very little other investigation of the factors that influence length of hospital stay of children and young people with Down syndrome, and we located no studies on risk of readmission or on emergency admissions of children and young people.

Without accurate information on live-birth and death rates, and hospital admission needs, it is not possible to plan for the health care support that children with Down syndrome and their families need.

This study's aims were to investigate the (1) incidence of live births of people with Down syndrome over a 25 year period, (2) frequency of deaths of children and young people with Down syndrome compared to matched controls, and (3) hospital admission frequency and

For peer review only - http://bmjopen.bmj.com/site/about/guidelines.xhtml

duration, emergency admissions, and readmissions of children and young people with Down syndrome compared to matched controls.

Methods

Ethical approval

Permission to access, link and analyse these data was granted by the Privacy Advisory Committee (PAC) to the National Health Service (NHS) National Services Scotland (NSS) and the Registrar General. Written informed consent was not obtained from participants for their clinical records to be used in this study, but patient records were anonymized and de-identified prior to analysis. Safehaven approval was granted to approved researchers to analyse the data.

Patient and Public Involvement

This study was undertaken by the Scottish Learning Disabilities Observatory, which has a specific remit for people with intellectual disabilities, including people with Down syndrome; its steering group includes partners from third sector organisations, including Down Syndrome Scotland. Results from this study will be disseminated for people with Down syndrome and their families/carers in easy-read version via the Scottish Learning Disabilities Observatory website and newsletters.

Study sample, setting, and process

All NHS Scottish Regional Genetics Centres (the East, North, South-East and West Scotland Centres) identified all live-birth infants screened positive for Down syndrome postnatally (live births with trisomy 21, mosaic trisomy 21, or unbalanced translocation resulting in

For peer review only - http://bmjopen.bmj.com/site/about/guidelines.xhtml

trisomy 21) between 01st January 1990 to 01st December 2015. In Scotland, everyone is given a unique Community Health Index (CHI) at birth which is used in their health records. The CHI database is held centrally by National Services Scotland (NSS), and was used to identify five controls without Down syndrome for each person with Down syndrome, matched on sex, age (by month and year of birth), and neighbourhood deprivation (using the Scottish Index of Multiple Deprivation: SIMD).[14,15] Down syndrome infants who could not be matched due to missing data or had less than one year of potential follow up time (i.e. births in 2015) were excluded from statisitcal analyses. The CHI also provides a means to record link each person identified with Down syndrome and their matched controls to routinely collected hospital admissions data (Scottish Morbidity Records 01: SMR01),[16] and National Records of Scotland death certificate data.[17] The end of the period of follow-up was 1st December 2015. However, children born in 2015 were excluded from the statistical analyses to allow a minimum potential follow up time of 1 year for all children.

Data sources and definitions

Live-births: Live births of babies with Down syndrome from all NHS Scottish Regional Genetics Centres, 1990-2015; Scottish live births in the whole population, from the National Records of Scotland births time series, 1990-2015 (NRS, 2019).[17]

Death: Deaths and causes of death by the International Classification of Diseases codes[18] according to death certificates registered at National Records of Scotland.

Hospital admissions: SMR01 contains episode-based records for all non-psychiatric, non-obstetric acute hospital admissions in Scotland. The information collected includes the date of admission to hospital and discharge from it and whether the admission was routine or emergency. In SMR01, continuous periods of care are accounted for with a Continuous Inpatient Stay (CIS) marker. This CIS marker ensures a series of individual episodes over an

For peer review only - http://bmjopen.bmj.com/site/about/guidelines.xhtml

unbroken period of care (e.g. transfers between wards or hospitals) can be identified and treated as one admission rather than several admissions. Transfer of a baby to a neonatal intensive care unit after birth is counted as a first admission. Data Quality Assurance Assessments for SMR01 are performed periodically.[19,20]

Admission type: Admission type is coded as emergency or routine. "Emergency" admissions are those which were unplanned in advance; planned admissions are "routine" admissions.

Birth year group: Grouped into the most recent cohort (2004-2014) and the least recent cohort (1990-2003).

SIMD: SIMD matching with controls and reporting is by quintiles, where SIMD 1 is the most deprived neighbourhoods, and SIMD 5 is the most affluent neighbourhoods. SIMD is calculated at datazone level, identified from post codes.

Discharge type: Discharge type is coded as a regular discharge or irregular discharge. Irregular discharges include e.g. a patient discharging himself/herself against medical advice, or death.

All follow up/censoring: Children were followed up from birth, and all models were censored on death or 1st December 2015 (whichever came first) unless stated otherwise.

Analyses

Incidence of Down syndrome: Incidence of Down syndrome births were calculated for each calendar year from 1990 to 2015 inclusive.

Death: Incidence rates for mortality per person time (per 1,000) were described, split by age group [for all ages, 0-1 month of life, from 1 - 12 months, from 13 - 60 months, from 61 - 120 months, from 121 - 180 months and from 181 - 240 months], as were underlying causes of death, for people with Down syndrome and their matched controls.

For peer review only

First hospital admissions: Incidence rates for first admissions per person time (per 1,000) for people with Down syndrome compared to controls were described, split by age group [for all ages, 0-1 month, from 1-12 months and from 13+ months]. Descriptive data for first admissions were presented including by sex, SIMD, year of birth, and duration of first admissions in days and this was compared using dependent t tests for continuous variables and chi square tests for categorical variables or mann-whitney U-tests for length of hospital stay. The relative risk of first hospital admission was compared for people with Down syndrome and controls using Stratified (by sex, birth year group, SIMD) Cox regression models (log-log plots confirmed the proportional hazards assumption was met). The follow up period was defined as from date of birth until date of first admission, and admission type was entered as a potential confounder.

Risk of emergency hospital admission: The relative risk of emergency first hospital admission was compared for people with Down syndrome and controls, analysed using Stratified (by sex, birth year group, SIMD) Cox regression models. The follow up period was defined as from date of birth until date of first admission.

Duration of hospital admission: Conditional (stratified by sex, birth year group, SIMD) linear regression was used to model the duration of first hospital admission (log transformed to help negate potential skewing of results) as the outcome, comparing Down syndrome and control groups. Admission type was entered as a potential confounder.

Risk of re-admission: The relative risk of readmission (the next admission post the first admission) was compared for people with Down syndrome and controls, analysed using Stratified (by sex, birth year group, SIMD) Cox regression models. The follow up period was defined as date of discharge from the first admission until date of the next admission. First admission type, and discharge type from first admission were entered as potential

For peer review only - http://bmjopen.bmj.com/site/about/guidelines.xhtml

confounders, and the interaction of group(Down syndrome versus controls)*admission type(emergency versus routine) was included.

Reference groups for all regression analyses were: not having Down syndrome, females, most affluent neighbourhoods, routine admissions, and regular type of hospital discharges. All analyses were conducted with IBM SPSS version 22.

Results

Between 01st January 1990 to 01st December 2015, the Scottish Regional Genetics Centres identified 1,479 live-births with Down syndrome. 1,235 were matched with 6,175 controls. 188 (12.7%) infants with Down syndrome were excluded from statistical analyses, as they could not be matched due to missing data on SIMD quintile (n=160) in view of missing post-code information, sex (n=21), month and year of birth (n=<5), and CHI e.g. due to migration out of Scotland (n=<5). A further 57 children were excluded as they were born in 2015 (and had less than 1 year of potential follow up time). Of the 1,479 infants born with Down syndrome, 689 (46.6%) were girls, 769 (51.9%) were boys, and sex was not recorded for 21 (1.4%). The proportion of births for the Down syndrome population, by the 5 SIMD quintiles were: 1= 21.3%, 2= 20.4%, 3=18.5%, 4= 20.1% and 5=19.7%, and SIMD data was missing for 160 (10.8%). Of the 1,235 infants with Down syndrome with matched controls, 591 (47.9%) were girls, 644 (52.1%) were boys. The proportion of births for the Down syndrome population, by the 5 SIMD quintiles were: 1= 21.0%, 2= 20.0%, 3=18.2%, 4= 19.8% and 5=19.9%. Hence they appear to be representative of all the Down syndrome infants that were born.

For peer review only - http://bmjopen.bmj.com/site/about/guidelines.xhtml

Incidence of Down syndrome live-births over time

Given the total number of infants born was 1,508,483, the incidence of Down syndrome live-births was 1,479/1,508,483 (1.0/1,000 births). Table 1 and figure 1 show the incidence of Down syndrome live-births for each year of the study period (with a moving average smoothed trend line to account for noise from year on year variation). The birth rate in Scotland has fallen since the early 1990s, whilst the incidence of Down syndrome shows some year-to-year variation and appears to have remained the same or risen since the early 1990s.

Table 1: Incidence of Down syndrome by year of birth

**Year of birth**	**Number of births registered in Scotland (all people)**	**Down Syndrome**	
	**N**	**N**	**(%)**
1990	65,973	60	0.09%
1991	67,024	66	0.10%
1992	65,789	38	0.06%
1993	63,337	35	0.06%
1994	61,656	43	0.07%
1995	60,051	46	0.08%
1996	59,296	40	0.07%
1997	59,440	72	0.12%
1998	57,319	54	0.09%
1999	55,147	52	0.09%
2000	53,076	60	0.11%
2001	52,527	61	0.12%
2002	51,270	44	0.09%
2003	52,432	58	0.11%
2004	53,957	79	0.15%
2005	54,386	63	0.12%
2006	55,690	44	0.08%
2007	57,781	70	0.12%
2008	60,041	63	0.10%
2009	59,046	73	0.12%
2010	58,791	59	0.10%
2011	58,590	63	0.11%

For peer review only - http://bmjopen.bmj.com/site/about/guidelines.xhtml

2012	58,027	59	0.10%
2013	56,014	58	0.10%
2014	56,725	62	0.11%
2015	55,098	57	0.11%*
**Total**	**1,508,483**	**1,479**	**0.10%**

*In 2015 the Down syndrome total is for 11 months only, so the % in 2015 is adjusted to 11/12 months in the general population

Deaths

Of the 1,235 people with Down syndrome with matched controls, 92 (7.4%) died during the study period (165,744 person months of follow up for the Down syndrome group), of whom 47 (51.0%) were female, 45 (48.9%) were male. Of the 6,175 matched controls, 23 (0.4%) died during the period (895,776 person months follow up for the controls), of whom 9 (39.1%) were females, and 14 (60.9%) were males. Death was therefore 18.5 times more common in the Down syndrome group. Most of the deaths of the people with Down syndrome occurred in infancy. Death incidence rates per person time (per 1,000) by age groups for the Down syndrome population versus controls were; 0.56 [CI 0.45-0.68] (n=92) versus 0.03 [CI 0.02-0.04] (n=23) for all ages, 17.81 [CI 9.87-32.17] (n=11) versus 0 for the 1st month of life, 3.87 [CI 2.96-5.07] (n=53) versus 0.17 [CI 0.09-0.29] (n=12) from 1 - 12 months, 0.36 [CI 2.96-5.07] (n=18) versus 0.02 [CI 0.01-0.05] (n=6) from 13 - 60 months, 0.15 [CI 0.07-0.32] (n=7) versus 0.01 [CI 0.00-0.04] (<5) from 61 - 120 months, 0.09 [CI 0.03-0.31] (n=<5) versus 0 from 121 - 180 months and 0 versus 0.02 [CI 0.00-0.08] (<5) from 181 - 240 months. The most common certified underlying causes of death for the Down syndrome group were congenital heart anomalies (n=33, 34.4%), Down syndrome (n=26, 27.1%) and infection (excludes respiratory infections) (n=11, 11.5%). This pattern was different compared to controls in whom causes of deaths were due to a range of other factors not found for people with Down syndrome (n=8, 34.8%), prematurity/perinatal causes (n=5, 21.7%) and congenital heart anomalies (n=5, 21.7%). Although less common than some

For peer review only - http://bmjopen.bmj.com/site/about/guidelines.xhtml

other causes, leukaemia was a cause of death among people with Down syndrome (n=<5) compared to controls where no deaths were from leukaemia.

Admissions

Table 2 presents details on the first all-cause hospital admissions for the two groups. 1,105/1,235 (89.5%) of the Down syndrome group had at least one hospital admission compared to 3,305/6,175 (53.5%) of the control group. Admission incidence rates per person time (per 1,000) by age groups for the Down syndrome population versus controls were; 72.89 [CI 68.72-77.32] (n=1,105) versus 40.51 [CI 39.15-41.92] (n=3,305) for all ages, 38.87 [CI 26.05-57.99] (n=24) versus 13.29 [CI 9.79-18.06] (n=41) for the first month of life, 56.44 [CI 52.03-61.23] (n=580) versus 24.94 [CI 23.62-26.33] (n=1,298) from 1-12 months, 117.47 [CI 107.62-128.22] (n=501) versus 74.55 [CI 71.32-77.92] (n=1,966) from 13+ months. Compared with the control group, the Down syndrome group were younger at the time of first hospital admission (median=0 months [IQR 0-300 months] versus 24 months [IQR 0-288]), had less admissions among females, were more equally spread across neighbourhoods whilst the control group had a gradient across SIMD groups with those in the most deprived areas having more hospital admissions, and had longer duration of admissions (5.03 days [median=2.00; IQR=1.00-4.00], versus 1.78 days [median=1; IQR=1.00-1.00]) by 3.25 days. The rate of admissions slightly increased over time for people with Down syndrome (from 528, 90.7% [in 1990-2003] to 577, 92.2% [in 2004-2014]) and decreased for controls (from 1,837, 63.3% [in 1990-2003] to 1,468, 44.8% [in 2004-2014]. The gap between the higher rate of admissions has widened over time for people with Down syndrome compared to controls.

For peer review only - http://bmjopen.bmj.com/site/about/guidelines.xhtml

Table 2: First all-cause hospital admissions characteristics

		**Down syndrome (n=1,105)**	**Control Group (n=3,305)**	**P-value+**
		N(%) Median(IQR)	N(%), Median(IQR)	
Age at admission (months)		0 (IQR 0-300)	24 (IQR 0-288)	<0.001
Sex	Male	572 (51.8%)	1,840 (55.7%)	<0.001
	Female	533 (48.2%)	1,465 (44.3%)	
Birth year group	Most recent	577 (92.2%)	1,468 (44.8%)	<0.001
	Less recent	528 (90.7%)	1,837 (63.3%)	
SIMD	Most affluent (5)	217 (19.6%)	570 (17.2%)	<0.001
	4	226 (20.4%)	609 (18.4%)	
	3	203 (18.4%)	591 (17.9%)	
	2	230 (20.8%)	720 (21.8%)	
	Most deprived (1)	229 (20.7%)	814 (24.6%)	
Duration of admission (days)		2.00 (IQR=1.00-4.00)	1.00 (IQR=1.00-1.00)	<0.001

IQR=Interquartile Range; SIMD=Scottish Index of Multiple Deprivation
Denominator for birth year group most recent/less recent are for Down syndrome n= 653/582/Control group n=3,275/2,900.

Emergency admission incidence rates per person time (per 1,000) by age groups for the Down syndrome population versus controls were; 56.78 [CI 53.13-60.72] (n=861) versus 28.88 [CI 27.73-30.07] (n=2,352) for all ages, 27.53 [CI 17.12-44.23] (n=17) versus 11.03 [CI 7.88-15.43] (n=34) for the first month of life, 48.66 [CI 44.57-53.11] (n=500) versus 20.42 [CI 19.23-21.69] (n=1,063) from 1-12 months, 80.66 [CI 72.57-89.65] (n=344) versus 47.71 [CI 45.14-50.42] (n=1,255) from 13+ months. Table 3 displays the results of the Cox regressions for time to first all-cause hospital admission. The risk of admission was higher amongst people with Down syndrome compared to controls, with an unadjusted hazard ratio (HR)=2.74 (95% CI 2.52-2.98). This remained after adjusting for type of first admission: HR=1.84 (95% CI 1.68-2.01).

Table 3: Risk for first all cause hospital admission

		**Separately entered**		**Simultaneously entered**	
		**HR value**	**95% CI**	**HR value**	**95% CI**
Group	No Down syndrome	ref	ref	ref	ref
	Down syndrome	2.74**	2.52-2.98	1.84**	1.68-2.01

For peer review only

For peer review only - http://bmjopen.bmj.com/site/about/guidelines.xhtml

First admission type	Routine	ref	ref	ref	ref
	Emergency	1.10*	1.00-1.20	1.19**	1.08-1.30

*P<.05; **P<.001
HR=Hazard Ratio; CI=Confidence Interval; SIMD=Scottish Index of Multiple Deprivation
Stratified models by sex, birth year group, SIMD

Table 4 displays the results of the Cox regressions for time to first emergency hospital admission. The risk of emergency admission was higher amongst people with Down syndrome compared to controls, with a hazard ratio (HR)=2.87 (95% CI 2.61-3.15).

Table 4: Risk for first emergency hospital admission

		**Separately entered**	
		**HR value**	**95% CI**
Group	No Down syndrome	ref	ref
	Down syndrome	2.87**	2.61-3.15

*P<.05; **P<.001
HR=Hazard Ratio; CI=Confidence Interval
Stratified models by sex, birth year group, SIMD

Duration of hospitalisation

The duration of first hospital admission was significantly longer for the Down syndrome group at 5.03 days [median=2 days] versus 1.78 days [median=1 day]) by 3.25 days. After adjustment (with a log transformed time variable, to help account for any data skew) for sex, birth year group, SIMD, and type of admission, the Coefficient=0.25 [CI 0.22-0.28] (table 5). This log transformed Coefficient can be interpreted as a ratio of difference for the Down syndrome population compared to the control group (exponentiate of the log transformed coefficient, exp(0.248)=1.28145993219 – 1 x 100=28, with a baseline length of stay (intercept) exp(0.12)=1.12749685158). This means the average length of stay was 28% higher in the Down syndrome population, compared to the controls. All exponentiated values are presented as percentages in table 5.

For peer review only - http://bmjopen.bmj.com/site/about/guidelines.xhtml

For peer review only

Table 5: Duration of first all-cause hospital admission (log transformed time variable)

		Separately entered			Simultaneously entered		
		**Coef**	**95% CI**	**Exp %**	**Coef**	**95% CI**	**Exp %**
Group	No Down syndrome	ref	ref		ref	ref	
	Down syndrome	0.24**	0.21-0.27	27%	0.25**	0.22-0.28	28%
First admission type	Routine	ref	ref		ref	ref	
	Emergency	0.02	0.00-0.04	2%	0.04**	0.02-0.06	4%

*P<.05; **P<.001

Coef=Coefficient; CI=Confidence Interval; Exp %=Exponentiated values as percentages

Stratified models by sex, birth year group, SIMD

Risk of re-admission

Of the Down syndrome group, 945/1,105 (85.5%) had at least one re-admission, compared to 1,685/3,305 (51.0%) of the control group. Re-admission incidence rates per person time (per 1,000) for the Down syndrome population versus controls were 54.85 [CI 51.46-58.46] (n=945) versus 15.06 [CI 14.36-15.80] (n=1,695). The baseline age at readmission was lower for the Down syndrome group with a median age of 6 months (IQR 1-17) compared to the controls with a median age of 11 months (IQR 5-20; P<.001). The unadjusted HR of Down syndrome for time to readmission following discharge from first admission was 2.44 [CI 2.13-2.79]. The HR when adjusted for type of admission, type of first discharge and group*emergency interaction was 2.56 [CI 2.08-3.14). Results are displayed in table 6.

For peer review only

For peer review only - http://bmjopen.bmj.com/site/about/guidelines.xhtml

Table 6: Risk of readmission following discharge from first admission

		Separately entered		Simultaneously entered	
		**HR value**	**95% CI**	**HR value**	**95% CI**
Group	No Down syndrome	ref	ref	ref	ref
	Down syndrome	2.44**	2.13-2.79	2.56**	2.08-3.14
First admission type	Routine	ref	ref	ref	ref
	Emergency	0.97*	0.83-1.13	1.39	1.05-1.84
Discharge type	Routine	ref	ref	ref	ref
	Irregular	0.86	0.83-0.89	0.85	0.82-0.88
Group*first admission type	-	-	-	1.06	0.78-1.44

*P<.05; **P<.001
HR=Hazard Ratio; CI=Confidence Interval
Stratified models by sex, birth year group, SIMD

Discussion

Principal findings and interpretation

We found the Scottish incidence of Down syndrome live-births to be 1.0/1,000 births over the last 25 years, with it being possibly higher now than in the early 1990s. The Scottish birth rate has fallen overall, but incidence of Down syndrome has not. Death rate was 18.5 times higher for the children/young people with Down syndrome than for their controls, and their causes of death differed, including causes that might be expected to have been amenable to good health care such as infections.

Over a 25 year period, we found that children/young people with Down syndrome compared with matched controls were considerably more likely to have had at least one hospital admission (IRR of 72.89 compared to 40.51; adjusted HR=1.84), and re-admissions (IRR of 54.85 compared to 15.06; adjusted HR=2.56). Their admissions were more likely to have been emergency admissions (IRR of 56.78 compared to 28.88; first emergency admission HR=2.87), and of longer duration 28% longer first admission after birth. Given the higher rate of first emergency admissions in the Down syndrome group, we included the interaction

For peer review only - http://bmjopen.bmj.com/site/about/guidelines.xhtml

For peer review only

of group x type of first admission (emergency or routine) in the analysis of readmissions, but they still remained more likely to be readmitted. We are not aware of any previous study of readmissions and emergency admissions in this population. The rate of admissions slightly increased over time (from 1990-2003 to 2004-2014) for the children/young people with Down syndrome (90.7% to 92.2%), whilst it decreased for the control group (from 63.3% to 44.8%), perhaps reflecting changes in medical practice over time, to keep children out of hospital as far as possible, but admitting for serious problems which the Down syndrome children may be more likely to experience. These findings are important, as they demonstrate the impact of additional health needs to both children and young people with Down syndrome, and to their families. This demonstrates the importance of statutory as well as informal support to families to avoid added problems in child development over and above the intellectual disabilities that the children with Down syndrome experience, and the impact caring can have on families. Additionally, the high rates of emergency admissions and re-admissions may indicate some poorer quality management of health care at the primary care level (to avoid admissions), and have not to our knowledge been reported before, though other factors, including underlying health conditions may contribute.

The Down syndrome group were younger at the time of first hospital admission, had a more equal sex distribution for first admission than the control group, and an equal distribution across extent of neighbourhood deprivation, whilst the control group had a gradient with those in the most deprived areas more likely to have had a hospital admission. This suggests that at present, the children with Down syndrome appear to receive comparable care in terms of first admission, regardless of their neighbourhood, and highlights that findings in the general population cannot be relied upon when planning services for people with Down syndrome/intellectual disabilities, such as focussing services in areas of greatest deprivation.

For peer review only - http://bmjopen.bmj.com/site/about/guidelines.xhtml

Families of children and young people with Down syndrome need focused support regardless of the areas they reside in.

Comparison with previous literature

As well as being 18.5 times more likely to die than their controls, we found that the children/young people with Down syndrome were more likely to be hospitalised, emergency hospitalised, re-admitted, and for longer durations. We have not identified any previous studies on re-admission with which to compare our findings, nor on emergency admissions. For the earlier period of 1977-2008 in Denmark, Zhu et al[13] reported that children and adults with Down syndrome had more than twice the rate of hospital admissions (all admissions, not separately reported for first admissions or repeat admissions), similar to our adjusted hazard ratios for first and repeat admissions, but we specifically investigated children and young people unlike that study. The smaller Australian study for the earlier period of 1983-2004 suggested the hospitalisation rate was five times higher, but relied on previously reported general population data from 1995.[12]

As our study shows, and as previously reported in a study of children with Down syndrome identified from population-based sources in 1997 (n=210) and 2004 (n=208) in Western Australia,[21] it is highly likely that admission rates change over time, so these two studies are not strictly comparable to ours, and we have found no studies contemporaneous with ours with which to draw comparisons. Previous, small scale studies reported length of admissions of children with Down syndrome to be 4.7 days in a Swedish study of 211 children followed up for at least 17 years,[22] and 7 days specifically for bronchiolitis in a small retrospective case-control study in Singapore,[23] compared with the 5.0 days we found. A further small

For peer review only - http://bmjopen.bmj.com/site/about/guidelines.xhtml

study of 213 children with Down syndrome aged 0-3 years in America reported high admission rates.[24]

The live birth incidence for Down syndrome we report is similar to that recently reported in England, USA, and Canada.[8-10] Antenatal screening changes may underlie the variability in the birth rates found over time. Previous research in Scotland showed that decisions to terminate pregnancy following antenatal diagnosis of aneuploidy had fallen over time,[1] which our results are also possibly suggestive of from the early 1990s to more recently. In contrast, in some other countries (e.g. Denmark), following the introduction of antenatal combined screening for Down syndrome in 2004, there was a concurrent increase in prenatal diagnoses and the number of Down syndrome live births decreased suddenly and significantly before then stabilising.[25] A higher proportion of the Scottish population have catholic faith compared with Denmark which might contribute in part to differences between countries: a range of complex factors influence women's decision-making regarding prenatal screening and diagnosis of Down syndrome, and it is possible that more societal acceptance of diversity and people with disabilities, or better availability of family support may influence decisions in Scotland over time,[1,26] although this requires further investigation.

Strengths and limitations

This is the first population-based study examining incidence of Down syndrome and health care utilisation over time in Scotland compared to the general population, and the first worldwide to investigate emergency hospital admissions and readmissions. It controlled for the potential confounders of age, sex, and SIMD. It was not subject to selection bias, as the population identification was based on routine data sources from all genetic service laboratories in Scotland (i.e. the whole population). The cox regression analysis for risk of

For peer review only - http://bmjopen.bmj.com/site/about/guidelines.xhtml

admissions and readmissions provides advantages over logistic or linear techniques as this includes consideration of time to event (person time at risk). However, there was some missing control data, mostly due to missing post-codes, and this is a limitation of the study. There was no data on ethnicity which is a limitation; the Scottish population is 96% white. The use of routinely collected hospital and death statistics has many advantages including large scale coverage, but there is likely to be a degree of coding inaccuracy which is not quantified. Children with Down syndrome have higher risk of comorbidities, so may be more likely to be transferred to neonatal intensive care units which might explain the increased risk of first hospital admission rates, but we could not distinguish such admissions in our dataset. We did not aim to investigate underlying conditions which are associated with admissions and death in the two groups.

Conclusions

As people with Down syndrome are living longer than in the past, we have provided contemporaneous statistics for the live birth rate of babies with Down syndrome and their childhood death rate compared with peers. We have found that their increasing survival is not without health consequences for the child, their family, and health services – we report the majority require hospital admissions, readmissions, and longer lengths of stays than their peers, which has received scant research attention in the past. Some factors may even suggest poorer health care than their peers, such as higher rates of emergency admissions, although we did not aim to investigate the conditions associated with admission. Together this brings important new findings necessary to address the challenge of planning support that children with Down syndrome and their families need. They require adequate health care, and also an awareness of the impact that hospitalisation can have on child development and family bonding. There may be a need to develop tailored packages of support for families with

For peer review only - http://bmjopen.bmj.com/site/about/guidelines.xhtml

children/young people with Down syndrome to cope with the higher health care needs of their offspring.

Footnotes

Contributors: LAH-M contributed to the study design, the analysis and interpretation of data, drafted the first article and revised it critically; DM, AH, LO contributed to study design, interpretation of data and revised the article critically; S-AC, JP, and RM conceived and designed the study, contributed to study analysis, interpretation of data and revised the article critically. All authors approved the final version

Funding: MRC Mental Health Data Pathfinder Award (MC_PC_17217), and the Scottish Government via the Scottish Learning Disabilities Observatory

Competing interests: The authors declare no competing interests

Ethical approval: Approval was gained from the Privacy Advisory Committee (PAC)

Data sharing statement: This study linked patient information held across administrative health datasets within ISD Scotland, with externally held data held by the Scottish Government and National Records of Scotland. Linkage and de-identification of data was performed by the Information Services Division (ISD) of NHS National Services Scotland (NSS). A data processing agreement between NHS NSS and University of Glasgow was drafted. The University of Glasgow were authorised to receive record linked data controlled and held by ISD within NSS, via access through the national safe haven. The ISD Statistical Disclosure Control Protocol was followed. It is therefore not possible to share data with other parties.

Reference

1. Jacobs M, Cooper S-A., McGowan R, Nelson S, Pell J. Pregnancy outcome following prenatal diagnosis of chromosomal anomaly: a record linkage study of 26,261 pregnancies. *PLoS ONE,* 2016; 11(12), e0166909.
2. Carothers AD, Boyd E, Lowther G, et al. Trends in prenatal diagnosis of Down syndrome and other autosomal trisomies in Scotland 1990 to 1994, with associated cytogenetic and epidemiological findings. *Genet Epidemiol.* 1999;16(2):179-90.
3. O'Leary L, Cooper SA, Hughes-McCormack L. Early death and causes of death of people with intellectual disabilities: A systematic review. *J Appl Res Intellect Disabil.* 2018;31(3):325-42.
4. Jensen KM, Bulova PD. Managing the care of adults with Down's syndrome. *BMJ.* 2014;349:g5596.
5. Hijii T, Fukushige J, Igarashi H, Takahashi N, Ueda K. Life expectancy and social adaptation in individuals with Down syndrome with and without surgery for congenital heart disease. *Clin Pediatr (Phila).* 1997;36(6):327-32.

For peer review only - http://bmjopen.bmj.com/site/about/guidelines.xhtml

6. Bull C, Rigby ML, Shinebourne EA. Should management of complete atrioventricular canal defect be influenced by coexistent Down syndrome. *Lancet.* 1985;1(8438):1147-9.
7. Wu J, Morris JK. The population prevalence of Down's syndrome in England and Wales in 2011. *Eur J Hum Genet.*2013;21(9):1016-9.
8. Morris, J.K., and Springett, A. (2014). The National Down Syndrome Cytogenetic Register for England and Wales: 2013 Annual Report. National Down Syndrome Cytogenetic Register. Queen Mary University of London.
9. de Graaf G, Buckley F, Skotko BG. Estimates of the live births, natural losses, and elective terminations with Down syndrome in the United States. *Am J Med Genet A.* 2015;167A(4):756-67.
10. Public Health Agency of Canada. Down syndrome surveillance in Canada, 2005-2013. Ottawa, 2017.
11. Ram G, Chinen J. Infections and immunodeficiency in Down syndrome. *Clin Exp Immunol.* 2011;164(1):9-16.
12. Fitzgerald P, Leonard H, Pikora TJ, Bourke J, Hammond G. Hospital admissions in children with down syndrome: experience of a population-based cohort followed from birth. *PLoS ONE.* 2013;8(8):e70401.
13. Zhu JL, Hasle H, Correa A, et al. Hospitalizations among people with Down syndrome: a nationwide population-based study in Denmark. *Am J Med Genet A.* 2013;161A(4):650-7.
14. National Statistics. Scottish Index of Multiple Deprivation 2012. Edinburgh: The Scottish Government; 2012.
15. Scottish Government, 2016: http://www.gov.scot/Topics/Statistics/SIMD)
16. Scottish Morbidity Records 01. http://www.ndc.scot.nhs.uk/Data-Dictionary/SMR-Datasets//SMR01-General-Acute-Inpatient-and-Day-Case/)
17. National Records of Scotland, (2019): https://www.nrscotland.gov.uk/statistics-and-data/statistics/statistics-by-theme/vital-events/births/births-time-series-data).
18. World Health Organisation.International Statistical Classification of Diseases and Related Health Problems, 10th Revision. Geneva: World Health Organisation, 1992.
19. Information Services Division (ISD) Scotland: Assessment of SMR01 Data 2010-2011. Scotland Report; May 2012
20. Turner M, Barber M, Dodds H, Dennis M, Langhorne P, Macleod MJ. Agreement between routine electronic hospital discharge and Scottish Stroke Care Audit (SSCA) data in identifying stroke in the Scottish population. *BMC Health Serv Res*. 2015 Dec 30;15:583.
21. Thomas K, Bourke J, Girdler S, et al. Variation over time in medical conditions and health service utilization of children with Down syndrome. *J Pediatr*. 2011;158:194–200
22. Frid C, Anneren G, Rasmussen F, Sundelin C, Drott P. Utilization of medical care among children with Down's syndrome. *J Intellect Disabil Res*. 2002;46:310–317
23. Ting TW, Chan HY, Wong PPC, Testoni D, & Lee JH. Down syndrome increases hospital length of stay in children with bronchiolitis. *Proceedings of Singapore Healthcare*. 2016; *25*(1), 64–67.
24. So SA, Urbano RC, Hodapp RM. Hospitalizations of infants and young children with Down syndrome: evidence from inpatient person-records from a statewide administrative database. *J Intellect Disabil Res.* 2007;51(Pt 12):1030-8.
25. Lou, S, Petersen, OB, Jørgensen, FS, Lund, ICB, Kjærgaard, S, Danish Cytogenetic Central Registry Study Group, et al. National screening guidelines and developments

For peer review only - http://bmjopen.bmj.com/site/about/guidelines.xhtml

in prenatal diagnoses and live births of Down syndrome in 1973–2016 in Denmark. *Acta Obstet Gynecol Scand* 2018; 97: 195– 203.
26. Olsen CL, Cross PK, Gensburg LJ. Down syndrome: interaction between culture, demography, and biology in determining the prevalence of a genetic trait. *Hum Biol.* 2003; 75(4):503-20.

For peer review only

Figure 1: Incidence of Down syndrome births by year in Scotland from 1990-2015

For peer review only - http://bmjopen.bmj.com/site/about/guidelines.xhtml

For peer review only

For peer review only - http://bmjopen.bmj.com/site/about/guidelines.xhtml

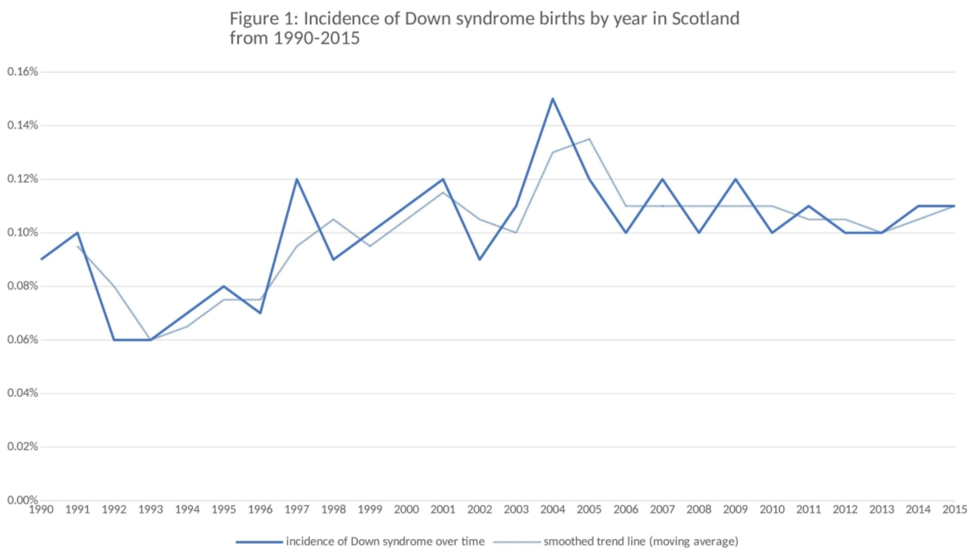

Figure 1: Incidence of Down syndrome births by year in Scotland from 1990-2015

338x190mm (300 x 300 DPI)

STROBE Statement—Checklist of items that should be included in reports of ***cohort studies***

	Item No	Recommendation	Page No
**Title and abstract**	1	(*a*) Indicate the study's design with a commonly used term in the title or the abstract<br>(*b*) Provide in the abstract an informative and balanced summary of what was done and what was found	2
**Introduction**			
Background/rationale	2	Explain the scientific background and rationale for the investigation being reported	4/5
Objectives	3	State specific objectives, including any prespecified hypotheses	5
**Methods**			
Study design	4	Present key elements of study design early in the paper	6
Setting	5	Describe the setting, locations, and relevant dates, including periods of recruitment, exposure, follow-up, and data collection	6-7
Participants	6	(*a*) Give the eligibility criteria, and the sources and methods of selection of participants. Describe methods of follow-up<br>(*b*) For matched studies, give matching criteria and number of exposed and unexposed	6-8
Variables	7	Clearly define all outcomes, exposures, predictors, potential confounders, and effect modifiers. Give diagnostic criteria, if applicable	6-8
Data sources/ measurement	8*	For each variable of interest, give sources of data and details of methods of assessment (measurement). Describe comparability of assessment methods if there is more than one group	6-8
Bias	9	Describe any efforts to address potential sources of bias	8
Study size	10	Explain how the study size was arrived at	6
Quantitative variables	11	Explain how quantitative variables were handled in the analyses. If applicable, describe which groupings were chosen and why	7-8
Statistical methods	12	(*a*) Describe all statistical methods, including those used to control for confounding<br>(*b*) Describe any methods used to examine subgroups and interactions<br>(*c*) Explain how missing data were addressed<br>(*d*) If applicable, explain how loss to follow-up was addressed<br>(*e*) Describe any sensitivity analyses	7-8
**Results**			
Participants	13*	(a) Report numbers of individuals at each stage of study—eg numbers potentially eligible, examined for eligibility, confirmed eligible, included in the study, completing follow-up, and analysed<br>(b) Give reasons for non-participation at each stage<br>(c) Consider use of a flow diagram	9-10
Descriptive data	14*	(a) Give characteristics of study participants (eg demographic, clinical, social) and information on exposures and potential confounders<br>(b) Indicate number of participants with missing data for each variable of interest<br>(c) Summarise follow-up time (eg, average and total amount)	9-14
Outcome data	15*	Report numbers of outcome events or summary measures over time	11-15

For peer review only

For peer review only - http://bmjopen.bmj.com/site/about/guidelines.xhtml

Main results	16	(*a*) Give unadjusted estimates and, if applicable, confounder-adjusted estimates and their precision (eg, 95% confidence interval). Make clear which confounders were adjusted for and why they were included<br>(*b*) Report category boundaries when continuous variables were categorized<br>(*c*) If relevant, consider translating estimates of relative risk into absolute risk for a meaningful time period	11-15
Other analyses	17	Report other analyses done—eg analyses of subgroups and interactions, and sensitivity analyses	11-15
**Discussion**			
Key results	18	Summarise key results with reference to study objectives	15-16
Limitations	19	Discuss limitations of the study, taking into account sources of potential bias or imprecision. Discuss both direction and magnitude of any potential bias	18
Interpretation	20	Give a cautious overall interpretation of results considering objectives, limitations, multiplicity of analyses, results from similar studies, and other relevant evidence	18-19
Generalisability	21	Discuss the generalisability (external validity) of the study results	18-19
**Other information**			
Funding	22	Give the source of funding and the role of the funders for the present study and, if applicable, for the original study on which the present article is based	19

*Give information separately for exposed and unexposed groups.

Note: An Explanation and Elaboration article discusses each checklist item and gives methodological background and published examples of transparent reporting. The STROBE checklist is best used in conjunction with this article (freely available on the Web sites of PLoS Medicine at http://www.plosmedicine.org/, Annals of Internal Medicine at http://www.annals.org/, and Epidemiology at http://www.epidem.com/). Information on the STROBE Initiative is available at http://www.strobe-statement.org.

For peer review only
